# A social cost-benefit analysis of meat taxation and a fruit and vegetables subsidy for a healthy and sustainable food consumption in the Netherlands

**DOI:** 10.1186/s12889-020-08590-z

**Published:** 2020-05-11

**Authors:** Marlin J. Broeks, Sander Biesbroek, Eelco A. B. Over, Paul F. van Gils, Ido Toxopeus, Marja H. Beukers, Elisabeth H. M. Temme

**Affiliations:** Centre for Nutrition, Prevention and Health Services, National Institute for Public Health and the Environment (RIVM), Antonie van Leeuwenhoeklaan 9, Bilthoven, 3721 MA The Netherlands

**Keywords:** Social Cost-Benefit Analysis, meat tax, fruit and vegetables subsidy, modelling, Netherlands, policy

## Abstract

**Background:**

Implementation of food taxes or subsidies may promote healthier and a more sustainable diet in a society. This study estimates the effects of a tax (15% or 30%) on meat and a subsidy (10%) on fruit and vegetables (F&V) consumption in the Netherlands using a social cost-benefit analysis with a 30-year time horizon.

**Methods:**

Calculations with the representative Dutch National Food Consumption Survey (2012–2014) served as the reference. Price elasticities were applied to calculate changes in consumption and consumer surplus. Future food consumption and health effects were estimated using the DYNAMO-HIA model and environmental impacts were estimated using Life Cycle Analysis. The time horizon of all calculations is 30 year. All effects were monetarized and discounted to 2018 euros.

**Results:**

Over 30-years, a 15% or 30% meat tax or 10% F&V subsidy could result in reduced healthcare costs, increased quality of life, and higher productivity levels. Benefits to the environment of a meat tax are an estimated €3400 million or €6300 million in the 15% or 30% scenario respectively, whereas the increased F&V consumption could result in €100 million costs for the environment. While consumers benefit from a subsidy, a consumer surplus of €10,000 million, the tax scenarios demonstrate large experienced costs of respectively €21,000 and €41,000 million. Overall, a 15% or 30% price increase in meat could lead to a net benefit for society between €3100–7400 million or €4100–12,300 million over 30 years respectively. A 10% F&V subsidy could lead to a net benefit to society of €1800–3300 million. Sensitivity analyses did not change the main findings.

**Conclusions:**

The studied meat taxes and F&V subsidy showed net total welfare benefits for the Dutch society over a 30-year time horizon.

## Background

There is a growing consensus that decreasing the environmental impact from food production and consumption are crucial to meet the Paris Climate Agreement and its goal to limit global warming [1, 2]. This is not surprising considering that agriculture and food production contribute an estimated 25% of total greenhouse gas (GHG) emissions [3]. Meat and dairy production is observed to be a disproportional contributor of emissions, attributing approximately half of food-derived GHG emissions, while only accounting for one-third of the dietary energy intake worldwide [1, 2, 4, 5]. Our dietary pattern not only affects our environment, it also influences our health [6]. A Western-type diet, characterized by a high red meat, processed meat, pre-packaged foods, fried foods, refined grains, and high-sugar drinks, has been strongly associated with non-communicable diseases (NCDs) such as cardiovascular diseases, diabetes, and cancer [1, 7, 8]. Consumption of red and processed meat exceeds recommended levels in most high and middle-income countries and has been associated with both negative health and environmental impacts [9, 10].

Recent modelling studies suggested that replacing meat with plant-based foods [8, 11], or selecting foods with low carbon footprints [11] reduce environmental impact and increase health. Nevertheless, the current Western diet is neither sustainable nor healthy [12–15]. The link between individuals’ environmental concerns as citizens and their behaviour as consumers were found to be quite weak and did not appear to influence meat-buying habits [16]. Consumers are not clear about healthy eating and everybody interprets it differently [17, 18]. This might even more apply to what it means to eat sustainable and environmentally friendly.

Governments may implement policy measures to stimulate healthy and sustainable choices. Systematic reviews demonstrate that subsidies to increase consumption of healthy foods and taxes to decrease consumption of unhealthy foods might be effective interventions in improving dietary behaviours and health [19, 20]. Similarly, modelling studies from various European countries predict taxes based on GHG emissions to be feasible to change dietary behaviours towards food groups with a lower environmental footprint [21–24]. Springmann et al. (2016) have estimated that the worldwide impact of taxing diet-related GHG emissions could result in a 9.6% decrease in GHG emissions originating from food production, while avoiding 500,000 deaths annually [25].

Price interventions may not only influence environment and health, but also have effects on other aspects of society, for example economic and distribution effects. A social cost-benefit analysis (SCBA) can incorporate these effects into a single analysis [26]. The SCBA is an instrument that can provide an overview of the (dis)advantages of measures, if possible quantified in euros and presented as a balance [26]. In this study, a SCBA was used to estimate and monetize the 30-year societal effects of a tax on meat and subsidization of fruit and vegetables (F&V) in the Netherlands.

## Methods

### SCBA framework

The essence of the SCBA framework is to estimate all (positive and negative) effects of a policy scenario on the total welfare of a population. A policy scenario is compared to a reference scenario without the policy but considering other autonomous trends in society. Various stakeholders (e.g. government and consumers) are identified in the society potentially affected by the policy (Supplemental file 1). An overview of the included societal effects and their indicators is presented in Table 1.

**Table 1:** Overview of the included societal effects and their indicators

Indicator	Model	Supporting Data
Consumption	Dynamo-HIA [27]	- Dutch National Food Consumption Survey [28]
		- Price elasticities [29]
		- More detail: supplemental file 2
Health	Dynamo-HIA [27]	Disease associations Dutch Health Council [30]
		- QALY value [26, 31]
		- Dutch Cost of Illness study [32, 33]
		- More detail: supplemental file 2
Productivity		- Labour participation [34–38]
		- Productivity (absenteeism and presenteeism [39])
		- More detail: supplemental file 1
Environmental impact	ReCiPe [40]	- Dutch National Food Consumption Survey [28]
		- Life Cycle Analysis (Blonk Consultants)
		- Extrapolations to all foods consumed (S.F 3)
		- Environmental indicator costs [41]
		- More detail: supplemental file 3
Consumer surplus		- Price elasticities [29]
		- More detail: supplemental file 1
Policy revenue		- Tax and subsidy
		- Value Added Tax (VAT)
		- More detail: supplemental file 1
Policy costs		- Implementation costs [42]
		- More detail: supplemental file 1
Stakeholders		- Consumers
		- Government

### Scenarios

Three scenarios were analysed within this study and compared to a reference (autonomous, no price change) scenario. Two taxation scenarios for total meat projected a 15% or 30% price increase at the consumer level whereas one scenario involved a subsidy on F&V resulting in a 10% price decrease. This manuscript does not discuss the way the price increase is implemented, e.g. via a CO_2_-tax, via a VAT in- or decrease of specific foods, or excise. The current Value Added Tax (VAT) in the Netherlands is 6% on foods but 21% on most other services and goods. The 15% meat tax is based on this transition. On all foods some level of VAT is required, therefore we arbitrarily selected the 10% discount for the fruit and vegetables scenario.

### Food consumption

Current food consumption in the Netherlands was obtained from the Dutch National Food Consumption Survey (DNFCS) 2012–2014 [28]. Food intake data from a representative sample of the population living in the Netherlands was collected between 2012 and 2014 on 2 non-consecutive days using 24 h dietary recalls. Only the consumption data for meat (red meat, processed meat, and poultry) and F&V were used for this study (Supplemental file 2). In the reference scenario, consumption changes over time were age and sex dependent but without an autonomous increasing or decreasing trend.

Price elasticities to calculate changes in consumption following a price change in the scenarios were obtained from a systematic literature review [29]. For total meat (red, white, and processed) the mean estimated price elasticity was − 0.60 (95% confidence intervals (CI): − 0.66; − 0.54) and for F&V -0.53 (95% CI: − 0.59; − 0.48). Consumption changes over time were calculated using the Dynamic Modelling for Health Impact Analysis (DYNAMO-HIA) model [27]. See for more model details the next section.

### Health impact assessment

The DYNAMO-HIA model was used to assess health impact of the scenarios [27]. DYNAMO-HIA is a Markov-type state-transition model and combines micro simulation of the risk factor and macro simulation of the disease and survival, using individual life tables with 1-year intervals to estimate developments in health over time. Boshuizen et al. describe the model in more detail [27] and Lhachimi et al. assessed the model’s validation [43]. New-borns and population size per given age and sex of the Netherlands derived from Statistics Netherlands were used as population input in the model.

Five diseases, diabetes type 2, stroke, lung cancer, coronary heart disease (CHD) and colorectal cancer, associated with meat and fruit and vegetables intake were assessed [30]. Disease incidence and prevalence of these five diseases in the Netherlands were included into the model based on Statistics Netherlands data of 2011. Disease disability and excess mortality weights of the model were used and were collected within the DYNAMO-HIA consortium in 2010.

Risk factor categories for the model were created using the relative risks (RR) of red and processed meat, and F&V consumption derived from a 2015 systematic literature review by the Dutch Health Council [30]. Since white meat (chicken and turkey) consumption is not associated with health it was not included in the health modelling.

Health effects were estimated by comparing the effects of the intervention compared to a reference scenario, in which no policy measures were implemented. Yearly differences in modelled chronic diseases and subsequent Quality Adjusted Life Years (QALYs) values between the reference and intervention scenarios were extracted from the model.

Uncertainty around the DYNAMO-HIA model estimates were evaluated using Monte Carlo simulations based on the 95% CI of the relative risk estimates, assuming a normal probability distribution. The 95% CI of 100 simulations per scenario are reported. Transition rates of risk factor categories were estimated using the method described by Van de Kassteele et al. [44]. The model presented increased or decreased health by calculating gained or lost QALYs. More details of the DYNAMO-HIA modelling are presented in Supplemental file 2.

### Environmental impact assessment

The environmental impacts of food were estimated with Life Cycle Analysis (LCA), a methodological tool to assess the environmental impact through the life cycle of a product (farm to plate principle). Supporting Life Cycle Inventories (LCI) data, individual unit processes in a supply chain, representative for Dutch market situations were provided by Blonk Consultants and were saved in SimaPro (version 8.52, PRe Consultancy B.V., Amersfoort, the Netherlands). Blonk consultants provided data on 225 foods in the Netherlands, covering approximately 80% of foods consumed in the DNFCS [28]. A panel of RIVM scientists performed extrapolations of the data to all foods consumed in the DNFCS 2012–2014. Environmental impact of the food products was then estimated using ReCiPe 2016 [45]. Environmental impact indicators that were estimated in the ReCiPe model were greenhouse gas emissions (kg CO_2_-eq), acidification (kg SO_2_-eq), eutrophication of salt (kg N-eq) and fresh (kg P-eq) water, and land use (m^2^a).

Efficiency gains in production of foods over time were estimated with the observed average change in GHG emissions intensity of the Dutch agro- and fishery industry [46]. Between 2000 and 2016, the relative intensity decreased by 20% (1.25% per year). This decrease was further linearly projected up to 2048 in the main analysis for all environmental impact indicators. See Supplemental Table 3 for more detail on the environmental impact assessment.

### Monetization of estimates

We applied both a value of €50,000 and €100,000 per QALY gained derived from the DYNAMO-HIA model and assumed the QALY value to remain stable over time, per Dutch guidelines [26, 31]. By using the QALY value in the main analyses, we assumed that consumers in their food choice decisions do not already value health aspects (informed consumers). Direct healthcare costs for diseases associated with consumption of meat and F&V were estimated using data from the Dutch Cost of Illness tool [47]. See for more details Supplemental file 1.

Environmental effects were monetized using the mean costs of GHG emissions (€0.057 per kg CO_2_-eq), acidification (€5.40 per SO_2_-eq), eutrophication of salt (€1.90 per kg N) and fresh (€3.11 per kg P-eq) water, and land use (€0.0261 per m^2^) estimated specially for the Dutch situation (see Supplemental file 3, [41]).

Within the SCBA framework, additional societal effects such as productivity, consumer surplus and tax income and subsidy expenses are also considered. The three components of productivity include absenteeism, presenteeism, and labour participation. Labour participation and productivity effects were estimated using the human capital method, according to Dutch guidelines [31]. The number of prevented cases of disease between 15 and 75 years old, the definition of the working population by Statistics Netherlands (CBS), was extracted from DYNAMO-HIA. To prevent double counting of effects, only income tax and welfare payment effects following changes in labour participation were considered, as shown by Koopmans et al. [31]. Productivity gains were estimated using the costs of absenteeism and presenteeism, which was estimated using absenteeism and presenteeism estimates of the modelled chronic diseases derived from Loeppke et al. [39].

For the meat scenarios, policy revenues were a combination of tax income minus the loss of VAT because of the reduced consumption. For the F&V scenario, these were the additional VAT benefits from an increased consumption minus the subsidy costs. The revenues were based on average cost per kg meat of F&V derived from CE Delft, adjusted yearly using the mean composite Consumer Price Index of the product category between 1996 and 2017 [34, 48].

Consumer surplus (CS), the welfare consumers derive from purchasing and consuming, was estimated by using the rule-of-half (RoH, see formula 1). The RoH approximates the changes in consumer benefits and is proscribed to estimate the changes in CS by Dutch SCBA guidelines [26]. The last societal effect was the estimated policy implementation costs. To account for time effects, in the main analyses a discount rate of 3% per year was used for all indicators (Supplemental file 1).

1 $$ \Delta CS=\frac{1}{2}\left(p0-p1\right)\ \left(q0-q1\right)\  where\ P= price\ and\ Q= quantity $$

### Sensitivity analyses

A one-way sensitivity analysis of the impact of different price elasticities on consumption and its related consequences was conducted. The price elasticity estimate was varied by adopting the upper and lower bound of the 95% confidence interval of the point estimate in assessing the change in consumption following a price intervention [29].

For the environmental impact calculations, a high and a low costs scenario were implemented. In the high costs scenario (High Environmental Costs-Low Efficiency Gains, HEC-LEG), the environmental impact indicators have high prices and in addition, the yearly efficiency gains in production were estimated to be low. In the low costs scenario, LEC-HEG, the opposite was estimated: low environmental impact costs at a high (1.75% per year) efficiency gain in production (Supplemental file 3).

Furthermore, the net welfare benefits were estimated when using a friction cost approach (instead of the human capital approach) to estimate productivity and participation. In addition, a perfect information scenario was calculated. In such a case, it is assumed that consumers consider and value all (known) costs, including (long-term) health before buying and consumption and therefore QALY gains or losses should not be considered. We also assessed the effect of changes in the discount rate used, by applying a rate of 1.5 and 4%, respectively [49].

## Results

### Food consumption

**Fig. 1:**
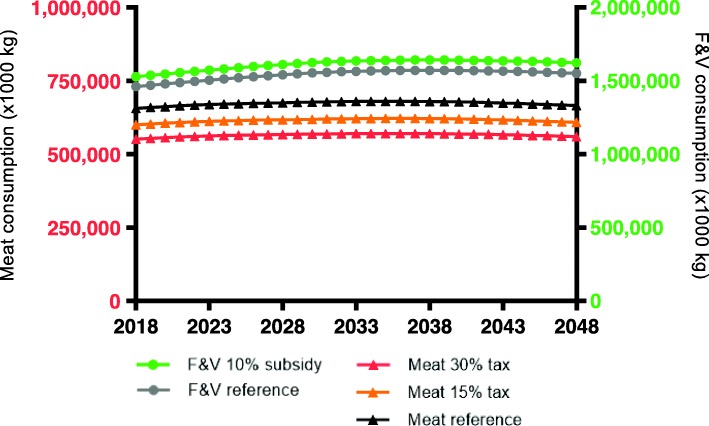
Modelled total Dutch consumption of meat and fruit and vegetables following food-pricing scenarios as compared to reference

In the reference scenario, in 2048, the total Dutch meat consumption is estimated to be 665,581,000 kg (kg). This translates to an average meat consumption of 39.2 kg per person per year or 107 g per day. A price increase of 15% or 30% is estimated to reduce the average meat consumption to 98.2 g per day in the 15% tax scenario and 90.3 g per day in the 30% tax scenario in 2048 (Fig. 1). In 2048, the estimated total F&V consumption is 1,551,853,000 kg or 250 g per day. Following the price decrease of 10%, the average consumption is estimated to increase to 261 g per day.

### Health impact assessment

**Fig. 2:**
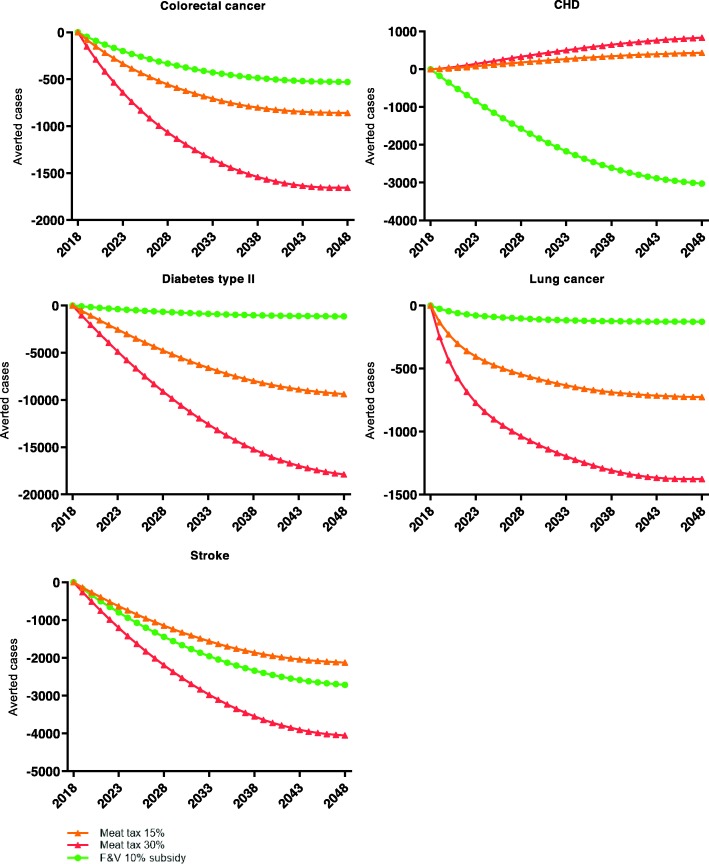
Modelled averted cases of associated chronic diseases following food-pricing scenarios compared to the reference scenario

Figure 2 presents the average modelled number of cases prevented per disease in the respective scenarios. In absolute numbers, a meat tax has the most impact on diabetes type 2 prevalence, with between 2093 and 15,449 (15% tax) or 5550–29,398 (30% tax) averted cases in the year 2048 (supplemental file 2). In the meat tax scenarios the incidence of CHD increases slightly, between 132 and 787 (15%) or 240–1506 (30%) additional cases, because of the reduction in the prevalence of the other four diseases. The F&V subsidy has most impact on stroke prevalence, between 1834 and 3586 averted cases in 2048. The number of QALYs gained in the year 2048, compared to the reference scenario is between 1119 and 3525 for the 15% meat tax scenario, 2122–6691 in the 30% meat tax scenario, and 1629–2483 in the 10% F&V subsidy.

### Environmental impacts

In the reference scenario, total environmental impact of meat consumption in 2048 is estimated to be approximately 15,225,000 ton CO_2_-eq (GHG emissions), 190,000 ton SO_2_-eq (acidification), 3000 ton P-eq (fresh water eutrophication), 33,000 ton N-eq (salt-water eutrophication), and 11,000 km^2^ (land use). In 2048, in the 15% taxation scenario, reductions in impact of 900,000 ton CO_2_-eq, 11,000 ton SO_2_-eq, 200 ton P-eq, 2000 ton N-eq and 750 km_2_ could be achieved. This is an 8.6% reduction for all impact categories over 30 years. In the 30% taxation scenario, the reduced consumption of meat could account for a 16% decrease in environmental impact compared to the reference scenario. In 2048, the estimated environmental impact of F&V consumption in the reference scenario is estimated to be 2000,000 ton CO_2_-eq (GHG emissions), 6000 ton SO_2_-eq (acidification), 200 ton P-eq (fresh water eutrophication), 1400 ton N-eq (salt water eutrophication), and 250 km^2^ (land use). The estimated higher consumption after a 10% subsidy of F&V could result in an increase of the environmental impact by 4.5% in 2048 (90,000 ton CO_2_-eq, 250 ton SO_2_-eq, 7 ton P-eq, 60 ton N-eq, and 11 km^2^).

### Social cost-benefit analysis

Total monetized effects over a 30-year period are presented in Table 2. In the 15% tax scenario, all benefits and losses lead to an overall net societal benefit between €3100 and 7400 million when a QALY value of €50,000 was applied. Introduction of a tax leading to a 30% price increase on meat-based products is estimated to result in overall benefits between €4000 and 12,300 million over 30 years. Subsidization of F&V is estimated to amount to an overall net societal benefit between €1800 and 3300 million.

**Table 2:** Total societal costs and benefits for all scenarios compared to reference over a 30-year period

Societal Effects	Scenario compared to reference (range)^**a**^		
	15% meat tax	30% meat tax	10% fruits and vegetables subsidy
Healthcare costs	€239 – 1613	€462 – 3081	€413 – 848
Health outcomes			
QALY €50,000	€834 – 2246	€1598 – 4289	€1043 – 1564
QALY €100,000	€1669 – 4492	€3196 – 8577	€2086 – 3127
Productivity	€313 – 1845	€604 – 3521	€473 – 1007
Environment	€3390	€6336	€−113
Policy revenue	€19,780	€36,334	€−9888
Consumer surplus	€−21,468	€−41,264	€9892
Policy costs	€−20	€−20	€−20
**Total welfare benefits (QALY €50,000)**	**€3069 – 7386**	**€4050 – 12,276**	**€1800 – 3289**
**Total welfare benefits (QALY €100,000)**	**€3904 – 9632**	**€5648 – 16,565**	**€2842 – 4853**

^a^Based on 100 iterations with the DYNAMO-HIA model using Monte Carlo simulations

Values are expressed in million 2018 euros

Stratifying the costs and benefits by consumers and governments, indicate that in the tax scenarios for consumers their positive health effects are outweighed by the loss in consumer surplus resulting in a net loss of welfare, especially in the 30% meat tax scenario (Table 3). However, in the F&V subsidy scenario, consumers benefit both from health gains as well as from an increased consumer surplus adding to their net social welfare. Because of the revenues of the tax and costs of the subsidy, the net benefits for the governments are in the tax scenario and net costs in the subsidy scenario.

**Table 3:** Societal costs and benefits for all scenarios compared to reference over a 30-year period stratified by consumers and government

Societal Effects	Scenario compared to reference (range)^**a**^					
	15% meat tax		30% meat tax		10% fruits and vegetables subsidy	
	Consumers	Government	Consumers	Government	Consumers	Government
Healthcare costs		€239 – 1613		€462 – 3081		€413 – 848
Health outcomes						
QALY €50,000	€834 – 2246		€1598 – 4289		€1043 – 1564	
QALY €100,000	€1669 – 4492		€3196 – 8577		€2086 – 3127	
Productivity	€41 – 253	€272 – 1592	€79 – 483	€525 – 3038	€78 – 158	€396 – 849
Environment		€3390		€6336		€−113
Policy revenue		€19,780		€36,334		€−9888
Consumer surplus	€−21,468		€−41,264		€9892	
Policy costs		€−20		€−20		€−20
**Total welfare benefits (QALY €50,000)**	**€–20,593 – −18,969**	**€23,661 – 26,355**	**€-39,587 – −36,492**	**€43,637 – 48,769**	**€11,013 – 11,614**	**€-9212 – −8324**
**Total welfare benefits (QALY €100,000)**	**€–19,758 – −16,723**	**€23,661 – 26,355**	**€-37.989 – −32.204**	**€43,637 – 48,769**	**€12,056 – 13,177**	**€-9212 – −8324**

Values are expressed in million 2018 euros

### Sensitivity analysis

**Fig. 3:**
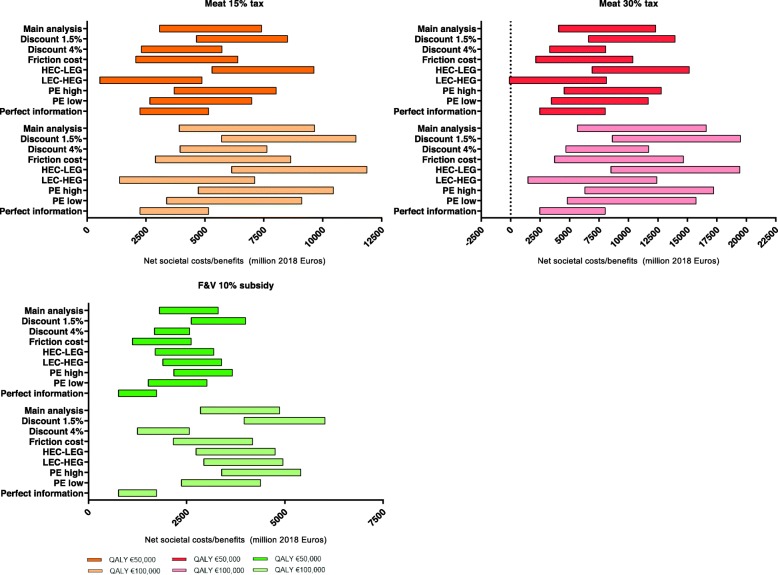
Net societal costs and benefits over a 30-year period of the sensitivity analyses of varying the discount rate, price elasticity and modelling methods as compared the no subsidy or tax scenario HEC-LEG: High environmental costs at low efficiency gain in production over time. LEC-HEG: Low environmental costs at high efficiency gain in production over time. Price elasticity high: − 0.66 for meat and − 0.59 for fruit and vegetables. Price elasticity low: − 0.54 for meat and − 0.48 for fruit and vegetables. Perfect information: gained or lost health because of consumption already accounted for by consumers; QALYs not considered in total welfare

Results of the sensitivity analyses are illustrated in Fig. 3. Except for one analysis, the minimum estimated net benefits for society remained positive. In the 30% meat tax scenario (with a QALY value of €50,000), in which the costs incurred for environmental impact indicators is low and food production systems have a high efficiency gain (1.75% per year) over time results in a net welfare between €-141–8085 million. In the sensitivity analyses involving a higher discount rate (4% instead of 3%), using the friction cost method instead of the human capital approach, lower price elasticities, or perfect information of consumers, the estimated benefits for society could be lower than observed in the main analyses (Fig. 3). In contrast, choosing a lower discount rate (1.5% instead of 3%), environmental impact indicators at a high cost level with a limited gain in production efficiency over time, and assuming higher price elasticities would be scenarios in which the estimated benefits could be higher compared to the main analyses.

## Discussion

A price increase for meat through a tax could lead to a net societal benefit for the Netherlands of about €3100–7400 million or about €4100–12,300 million for respectively a 15 or 30% tax over a period of 30 years. A price decrease of F&V by means of a subsidy could lead to a net societal benefit of about €1800–3300 million. Important contributors to net welfare gains or losses are consumer surplus and policy revenues/costs. Several assumptions, such as estimated costs of environmental impact indicators and production efficiency gains over time, as well as selected discount rate or using the informed consumers’ assumption were shown to have a large impact on the estimated results. However, in almost all sensitivity analyses the total estimated effect was still a net welfare benefit for society.

A SCBA aims to take into account all types of costs and benefits of interventions, irrespective of which stakeholders win or lose from the policy scenarios. However, it is also important to show the distribution of these benefits and costs over the different parties, especially when the losses are financial while the gains are non-financial, such as a gain in QALYs. In the tax scenarios, consumers could be net payers because of the loss of consumer surplus, whereas the government could gain income from the tax revenues. For the subsidy scenario, this is the other way around.

Even though no study as of yet has estimated the total societal effects of a tax on meat or subsidies on F&V, various studies have assessed the effects on consumption, health or environment separately. Mhurchu et al. estimated a 2% decrease in all-cause mortality following a 20% subsidy on F&V in a modelling study in New Zealand [50]. In Sweden, Sall and Gren estimated a variable environmental tax (9–30%) on meat and dairy to decrease GHG emissions by 12%, at a specific point in time [21]. Briggs et al. applied a tax of £2.72/ton carbon dioxide equivalents/ 100 g product applied to all food and drink groups with above average GHG emissions in the United Kingdom [51]. They estimated GHG emissions reductions up to 18,683,000 ton CO_2−_eq per year compared to the current situation, while saving 7700 lives per year. In our current study, introduction of a 15% tax on meat could reduce GHG emissions by 3,600,000 ton CO_2_-eq in 2048 if our population size were similar to the UK, which population is around four times larger. This might be an indication that taxing of all foods with above average GHG emissions is more effective than singling out only meat.

In a recent paper, Springmann et al. estimated the global and national health care costs related to meat consumption and calculated from this the price of meat if all health effects were incorporated in the price [52]. In Western countries, such as the Netherlands, the increase in price should be 21.3% for red meat and 111.2% for processed meats.

In addition to integrating health in the price of foods, also environmental and social factor could be added to calculate the ‘true price’ of a food [53]. Evidently, as Springmann et al. described that integrating health would already add 20% (red meat) and more than 100% (processed meat) to the price, integrating these other components would add to this even more. From a societal perspective, in the current analyses, it showed that the meat tax scenarios of 15% or 30% could result in net benefits for the society. Although it is clear, that not all health and environmental costs are then covered.

The analyses focus on the Dutch consumption, but the global perspective used in the article of Springmann et al. indeed is important to take into account. When price policies are applied only nationally, there will be trading effects with economic consequences that are yet not included in the current analysis. In addition, foods and feed are imported from other regions. International or European agreements will be important to prevent the possibility of carbon leakage, in which the emission reduction by one country is followed by an increase in another country, as environmental effects are not confined to national borders [23, 54]. The used environmental impact is based on average Dutch market (including import) food consumption data [40] and their monetized values are not specific for the agro sector [41]. For monetized local environmental effects of Dutch foods this might be a good estimation, but some of our consumed foods and feed are imported and the associated environmental impact and associated costs are located in other countries [55].

Changing prices of food products will likely have diverse effects on groups within society, in particular regarding socio-economic status (SES). While the environmental impact of food consumption is similar between SES groups, low SES groups generally have an unhealthier diet and tend to eat less F&V and fish and more meat and fats compared to higher SES groups [28, 55]. A recent Dutch study indicates that the socioeconomic differences in healthiness of the diet are likely to further increase [55]. As low SES groups have been observed to be more responsive to the price of food, price intervention policies could be an effective approach to increase healthiness and environmental friendliness of diets [29]. A combined taxation on meat and a subsidy on F&V could compensate some of the losses felt especially in the low SES groups. The current model did not allow a combined calculation of the subsidies and taxes and for stratification by SES, which is predicted to be more effective and result in higher societal benefits than single interventions. In addition, more research is needed to estimate cross-price elasticities by SES as well: which foods items will be consumed more often to replace the more expensive meats.

Discouraging meat consumption is highly related to current consumption levels and disease profiles of countries [10]. Although linked to adverse health effects on noncommunicable diseases, such as colorectal cancer and cardiovascular diseases at high consumption levels, meat is a nutrient-dense food in itself. In developing countries, because of the very low meat consumption, an small increase of meat consumption could help to alleviate some of the micronutrient deficiencies [10, 52]. It should also be noted that sustainable diets are only a part of the solution towards a more sustainable consumption pattern [3]. Using renewable energy sources, recycling waste, using trains instead of airplanes are, among others, additional steps for a sustainable future [56].

This study has a number of strengths. The consumption data was from a representative sample of the Dutch population [28]. Estimated total Dutch consumption of meat at baseline is in line with other reports on Dutch consumption of 77 kg meat (excluding bones 38 kg [57]). Relative risks related to food consumption are supported by consistent and strong evidence derived from a systematic literature review [30]. The DYNAMO-HIA model allows dynamic simulations and used Dutch data as input with an integration of the range of the estimates. Additionally, the used environmental data were obtained from 2018 LCA analyses tailored for the Dutch food consumption context. The SCBA framework allows for comparison between different fields such as health, environmental and other societal effects by monetizing the effects allowing us to estimate the societal impact of taxes on meat or subsidies on F&V. The Dutch guidelines on SCBA analyses in the health and environmental domains were followed [26, 31, 49]. Finally, several multiple sensitivity analyses were used to demonstrate the effect of different input parameters and methods on the net societal benefit.

Like any modelling study, the current study also has limitations to consider. Firstly, in the model, cross-elasticities could not be used to consider replacements following a decrease in meat consumption. Replacement foods could be healthy and sustainable, such as F&V, but also have high environmental impacts or negative health effects, such as cheese. In addition, the DYNAMO-HIA model is limited to one risk factor, and thus does not allow for analysis of a combination scenario of a meat tax and F&V subsidy. A replacement food may also partly compensate for the lost consumer surplus experienced in the meat tax scenario. Secondly, when considering the consumption in the reference scenario, meat and F&V consumption were assumed similar over time per age and sex category. An autonomous trend in food consumption patterns was not included, as there was no conclusive evidence available about the changes in meat and F&V consumption in the Netherlands [28, 55, 57]. In addition, whether the number of vegetarians/vegans would increase following an increase in price of meat-based products or a decrease in price of F&V could not be predicted. Thirdly, the health modelling was performed using categorization of food consumption. As the observed effects in food consumption were relatively small, the categorization might lead to an underestimation of the true effect on health when the change is not large enough to be placed in another consumption category, while any change in food patterns, even small ones, may have positive effects for health and environment at population level. Fourthly, the used SCBA framework uses monetization of effects in order to estimate societal benefits or costs, comparing a scenario with a reference scenario. In the SCBA, consumer surplus was one of the largest contributors to the balance in both the tax (as costs) and subsidy (as benefits) scenarios. Although utility euro’s and not financial euros, consumer surplus is to be considered in a SCBA according to the Dutch guidelines [26]. Another weakness is that this study has only estimated effects on the food consumption side, and not the food production side. For the tax on meat-based products, increases in export following a lower domestic demand for meat products could offset some environmental gains in terms of national GHG emissions and may worsen the financial situation for some livestock farmers. If not implemented in Europe, Dutch consumers might also increase spending across border to avoid the higher prices. With the Danish fat tax, however, this effect was observed to be relatively small [58].

The attention for healthiness and sustainability of food consumption grows [59]. Information like derived in this SCBA could prove crucial to accurately estimate long-term effects of new policies. To improve assessment of societal effects of price interventions, real-life assessment of price changes could prove highly informative in addition to price elasticities of demand. Virtual supermarkets such as those studied by Waterlander et al. [60, 61] can potentially increase knowledge about consumer behaviour following price changes, such as introduction of taxes and/or subsidies. Alternatively, discrete choice experiments (DCE) and/or willingness-to-pay (WTP) assessments could be used for studying healthier and more sustainable foods choices.

## Conclusions

The presented results demonstrate that a reduction in chronic disease prevalence, from reductions in meat consumption or increase in F&V consumption, is leading to benefits to society following gains in quality of life, mortality, healthcare spending, and productivity. In terms of environmental impact, a reduction of 8.5 and 16% in the 15 and 30% tax scenario respectively but an increase of 4.6% in the subsidy scenario was estimated. Concluding, a 15% or 30% tax on meat or a 10% subsidy on F&V could lead to net welfare gains for the Dutch society.

## Supplementary information

**Additional file 1.** Social Cost-Benefit Analysis.

**Additional file 2.** Health impact assessment.

**Additional file 3.** Estimating environmental impact of dietary changes.

## Data Availability

The datasets used and/or analysed during the current study are available from the corresponding author on reasonable request. The DYNAMO-HIA model is freely available from https://www.dynamo-hia.eu/nl.

## Abbreviations

CI: Confidence interval
CS: Consumer surplus
DCE: Discrete choice experiment
DNFCS: Dutch National Food Consumption Survey
DYNAMO-HIA: Dynamic Modelling for Health Impact Analysis
F&V: Fruit and vegetables
GHG: Greenhouse Gas
HEG-LEG: High Environmental Costs-Low Efficiency Gains
LCA: Life Cycle Analysis
LCI: Life Cycle Inventories
LEG-HEG: Low Environmental Costs-High Efficiency Gains
NCD: Non-communicable diseases
QALY: Quality Adjusted Life Year
RR: Relative risk
SCBA: Social cost-benefit analysis
SES: Social economic status
VAT: Value added tax
WTP: Willingness to pay
