# Supplementary material for: A social cost-benefit analysis of meat taxation and a fruit and vegetables subsidy for a healthy and sustainable food consumption in the Netherlands
Source: BMC Public Health. 2020 May 11;20:643. doi: 10.1186/s12889-020-08590-z (PMC7212616; doi:10.1186/s12889-020-08590-z)
Supplement: Supplementary file 1 — Additional file 1. Social Cost-Benefit Analysis. [file 12889_2020_8590_MOESM1_ESM.docx]

**Supplemental file 1. Social Cost-Benefit Analysis.**

Price interventions can have ambiguous effects on welfare by introducing unwanted consequences from an ethical, economic or social point of view (1). By using a societal perspective, we aim to provide an integral estimate of the effects of implementing a tax on meat-based products or subsidization of fruits and vegetables. We followed the guidelines for a Social Cost-Benefit Analyses (SCBA) for the Dutch context (2-4). We estimated effects of such interventions on health, environment, productivity, price intervention cost or revenue, changes in VAT revenue and implementation costs. We performed sensitivity analyses to assess robustness of the main analysis.

**Healthcare cost**

We applied both a value of €50.000,- and €100.000,- per Quality Adjusted Life Year (QALY) gained derived from the DYNAMO-HIA model and assumed the QALY value to remain stable over time, per Dutch guidelines (2, 3). We assessed the value of the total QALYs gained in a scenario compared to the reference scenario per year and applied a discount rate of 3%. The net present value was given by the sum of monetized QALYs in 2018 euros. Additionally, we applied a rate of €100.000,- per QALY (3). To estimate direct healthcare costs, we used the Dutch Cost of Illness study, using the average costs of treatment per patient from the NIVEL Netherlands Primary Care Database: General Practitioners. (5, 6) We present monetized estimations in **Table 1**. The QALY values of 100,000 are simply twice the benefits of the QALY €50,000 section.

**Table 1.** Total health cost-benefits of the three scenarios compared to reference scenario. All values are in million 2018 euros.

|  | **Compared to reference scenario^1^** | | |
| --- | --- | --- | --- |
|  | Scenario 15% tax (range) | Scenario 30% tax (range) | Scenario 10% subsidy (range) |
| QALY value €50.000 | €834 - €2246 | €1,598 - €4,289 | €1,043 - €1,564 |
| Direct healthcare | €239 - 1,613 | €462 - 3,081 | €413 - 848 |
| **Net health** | **€1,073 - 3,859** | **€2,059.84 - 7,369.34** | **€1,455 - 2,412** |

*^1^Range based on 95% confidence interval based on 100 iterations of the model using Monte Carlo simulations.*

**Productivity**

Labour participation and productivity results are presented in **Tables 2 and 3**. Per chronic disease, prevented cases between 15-75 years old, the definition of the working population in the Netherlands by Statistics Netherlands (CBS), were extracted from the DYNAMO-HIA model to estimate the change in labour participation. We applied the human capital method, according to the Dutch guidelines (3). A friction cost approach was applied as a sensitivity analysis (see sensitivity analysis). Labour participation data for diabetes type 2 was derived from a Dutch study on diabetes in 2007 (7). Labour participation for lung cancer and colorectal cancer were obtained from a 2012 Dutch study (8). Labour participation data of coronary heart disease (CHD) was derived from a 2007 Dutch study into labour participation by TNO (9). Finally, participation data of stroke patients was derived from an international systematic literature review due to lack of national data (10). We estimated the effect of changes in labour participation on governmental income tax using an average tax rate of 34% and the cost of one full-time equivalent (FTE) of 2017, which is €57,646.21 (11). To assess the difference in welfare payments from decreased unemployment due to chronic disease, we used the welfare subsidy for a single person in 2017, and assumed half of the welfare payments to be paid, per guidelines (11). To account for rising wages, we increased the yearly cost of an FTE by 1.8%, the average growth of Dutch wages from 2001-2017 (11). Welfare payments were increased yearly by 1.05%, the 20 year average growth factor in the Netherlands, as obtained from Eurostat (12).

Productivity gains were estimated using the averted costs of absenteeism and presenteeism. We present the results in **table 3**. Absenteeism and presenteeism data for diabetes type 2, colorectal cancer, lung cancer and CHD were derived from a large multiemployer study by Loepkke et al (13). As there was no data on presenteeism of stroke patients, we used fatigue as proxy derived from Loepkke et al, as studies show stroke patients to be heavily affected by fatigue when returning to and during work (13-15). To monetize the change in productivity, we used the average cost of labour per day in the Netherlands derived from the CBS and used the human capital approach. We applied a 3% discount rate per year to account for time preferences, per Dutch guidelines and increased the cost of labour per day by 1.8% per year, the average growth of Dutch cost of labour per day from 2001-2017 (3, 11).

**Table 2.** Labour participation compared to reference scenario per modelled disease. All values are in million 2018 euros.

|  | **Scenario compared to reference scenario** **(range)^1^** | | |
| --- | --- | --- | --- |
| Participation | Scenario 15% meat tax | Scenario 30% meat tax | Scenario 10% fruit and vegetables subsidy |
| Diabetes type 2 | €247.9 - €1,259.3 | €475.7 - €2,402.2 | €30.7 - €167.8 |
| Colorectal cancer | €-2.3 - €40.7 | €-4.1- €78.1 | €6.7 - €15.8 |
| Lung cancer | €9.3 - €26.5 | €18.1 - €50.2 | €2.1 - €4.4 |
| Stroke | €78.1 - €277.9 | €150.1 - €530.6 | €152.4 - €300.1 |
| Coronary heart disease | €-60.6 - €12.6 | €-115.0 - €23.4 | €203.8 - €360.7 |
| **Total labour participation** | **€272.4 - €1,591.6** | **€524.7 - €3,037.7** | **€395.8 - €849.0** |

*^1^Range based on 95% confidence interval based on 100 iterations of the model using Monte Carlo simulations.*

**Table 3**. Productivity compared to reference scenario per modelled disease. All values are in million 2018 euros.

|  | **Scenario compared to reference scenario** **(range)^1^** | | |
| --- | --- | --- | --- |
| Productivity | Scenario 15% meat tax | Scenario 30% meat tax | Scenario 10% fruit and vegetables subsidy |
| Diabetes type 2 | €24.9 - €126.5 | €47.9 - €241.4 | €3.1 - €16.9 |
| Colorectal cancer | €-2.2 - €39.6 | €-4.0 - €76.1 | €6.7 - €15.4 |
| Lung cancer | €9.4 - €25.9 | €18.0 - €49.1 | €2.1 - €4.3 |
| Stroke | €18.0 - €62.8 | €34.5 - €120.0 | €35.0 - €67.8 |
| Coronary heart disease | €-9.1 - €-1.9 | €-17.2 - €-3.5 | €30.7 - €53.5 |
| **Total labour participation** | **€41.0 - €235.0** | **€79.2 - €483.2** | **€77.7 - €158.0** |

*^1^Range based on 95% confidence interval based on 100 iterations of the model using Monte Carlo simulations.*

**Price intervention revenue and consumer surplus**

Governmental revenues generated by taxation of meat products and cost of subsidizing fruit and vegetable products were estimated using a weighted average price. Weights of meat-based product groups were based on Dutch meat consumption between 2005-2016, compiled by Wageningen Economic Research (16). Price intervention revenues were calculated using yearly consumption derived from the DYNAMO-HIA model following the price intervention. To account for inflation, we adjusted the prices using the average Consumer Price Index from 1996-2017. We estimated the tax revenue by using formula (1), in which *q_1_* is the adjusted consumption following price intervention, and *p_1 –_ p_0_* is the change in price due to a tax or subsidy.

1. *Revenue = q*1*(p*1 - *p*0)

To assess the change in consumer utility, we estimated the change in consumer surplus (CS) using the rule-of-half (RoH), formula (2). The RoH approximates the changes in consumer benefits, which includes the change in surplus due to change in price for current consumers and change in welfare. An important assumption of the RoH is linearity of the demand function, which is assumed to be the case when price changes are not too large (17). The RoH uses uncompensated demand curve, and the price effect is equal to the sum of the substitution effect and income effect.

1. *ΔCS = ½(p1 - p0) (q1 - q0)*

We applied a 3% discount rate per year on both the difference in CS and policy revenues or cost.

*VAT revenue and policy implementation costs*

Changes in consumption will affect governmental revenue derived from value-added tax (VAT). We quantify this effect by assessing the change in consumption between the scenario and the reference scenario and applying a 6% tax rate to the value of the change, the VAT rate in 2018 for food products in the Netherlands. From 2019 onwards, the new VAT is 9%. Policy implementation costs, such as costs to the tax authorities, changing prices in cashier systems and other IT systems were estimated using the same method as the SCBA by De Wit et al (18). Results are presented **in table 4**.

**Table 4.** Price intervention cost, difference in VAT revenue compared to reference scenario and implementation costs over 30 years. All values are in million 2018 euros.

|  | **Scenario compared to reference** | | |
| --- | --- | --- | --- |
| **Effects** | **15% meat tax** | **30% meat tax** | **10% fruit and vegetables subsidy** |
| VAT revenue | €-725.8 | €-1,335.3 | €222,6 |
| Policy implementation | €-20.0 | €-20.0 | €-20.0 |
| **Net policy revenue** | **€-745.8** | **€-1,355.3** | **€202,6** |

**Sensitivity analysis**

We performed one-way and scenario sensitivity analyses. We assessed the effect of a friction cost method for productivity and labour participation using a friction period of 85 days, the current friction period used in Dutch health economic guidelines (**Table 5**, (19)). We varied the price elasticity to the lower and upper bound of the 95% confidence interval (**Table 6a-f**). As scenario analysis, we assessed the effect of a 1.5% and a 4% discount rate (**Table 7a-c, 8a-c**)**. Table 9** is a summary table of the effects of the sensitivity analyses on the net societal costs and benefits as presented in Figure 3 in the main article.

***Friction cost approach***

**Table 5.** Friction cost method to estimate labour participation and productivity. All values are in million 2018 euros.

|  | **Scenario compared to reference** | | |
| --- | --- | --- | --- |
|  | Scenario 15% tax | Scenario 30% tax | Scenario 10% subsidy |
| Productivity | €3.2 | €6.2 | €3.5 |
| Labour participation | €40.8 | €75.3 | €23.7 |
| **Total** | **€44.0** | **€81.5** | **€27.2** |
| **Difference with human capital** | **€-996.7** | **€-1,911.3** | **€-678.4** |

***Price elasticity***

**Table 6a.** Sensitivity analysis with main analysis (reference) and lower and upper bound of price elasticity in the 15% tax scenario. All values are in million 2018 euros.

| **Meat - Scenario 15% tax** | **Reference** | **Lower bound PE** | **Upper bound PE** |
| --- | --- | --- | --- |
| Tax revenue | €20,506.2 | €20,663.4 | €20,288.9 |
| Consumer surplus | €-21,467.9 | €-21,549.1 | €-21,359.3 |
| VAT | €-725.8 | €-668.4 | €-807.8 |

**Table 6b.** Sensitivity analysis with main analysis (reference) and lower and upper bound of price elasticity in the 30% tax scenario. All values are in million 2018 euros.

| **Meat - Scenario 30% tax** | **Reference** | **Lower bound PE** | **Upper bound PE** |
| --- | --- | --- | --- |
| Tax revenue | €37,669.3 | €38,158.6 | €37,065.6 |
| Consumer surplus | €-41,264.3 | €-41,514.1 | €-40,962.5 |
| VAT | €-1,335.3 | €-1,266.3 | €-1,470.5 |

**Table 6c.** Sensitivity analysis with main analysis (reference) and lower and upper bound of price elasticity in the 10% subsidy scenario. All values are in million 2018 euros.

| **Fruit vegetables**  **10% subsidy** | **Reference** | **Lower bound PE** | **Upper bound PE** |
| --- | --- | --- | --- |
| Tax revenue | €-10,110.7 | €-10,063.5 | €-10,129.7 |
| Consumer surplus | €9,892.2 | €9,855.5 | €9,888.7 |
| VAT | €222.6 | €211.9 | €245.6 |

**Table 6d.** Sensitivity analysis with main analysis (reference) and lower and upper bound of price elasticity on health gains (Direct healthcare and QoL). All values are in million 2018 euros.

|  | **Reference** | **Lower bound PE** | **Upper bound PE** |
| --- | --- | --- | --- |
| Scenario 15% tax | €2,440.8 | €2,247.3 | €2,725.1 |
| Scenario 30% tax | €4,681.3 | €4,327.6 | €4,945.2 |
| Scenario 10% subsidy | €1,910.3 | €1,671.4 | €2,175.2 |

**Table 6e.** Sensitivity analysis with main analysis (reference) and lower and upper bound of price elasticity on productivity.

|  | **Reference** | **Lower bound PE** | **Upper bound PE** |
| --- | --- | --- | --- |
| Scenario 15% meat tax | €141.9 | €130.7 | €172.6 |
| Scenario 30% meat tax | €271.8 | €250.8 | €285.9 |
| Scenario 10% subsidy | €113.0 | €104.2 | €128.7 |

**Table 6f.** Sensitivity analysis with main analysis (reference) and lower and upper bound of price elasticity on participation.

|  | **Reference** | **Lower bound PE** | **Upper bound PE** |
| --- | --- | --- | --- |
| Scenario 15% meat tax | €898.7 | €831.0 | €1,017.8 |
| Scenario 30% meat tax | €1,721.0 | €1,594.4 | €1,819.1 |
| Scenario 10% subsidy | €592.5 | €544.3 | €680.3 |

***Discount rate sensitivity***

**Discount rate 1.5%**

**Table 7a.** Total welfare effect with discount rate of 1.5% – 15% meat tax. All values are in million 2018 euros.

| **Scenario 15% meat tax** | **Confidence interval low** | **Confidence interval high** |
| --- | --- | --- |
| Health - Low QALY | €1,064.1 | €2,899.0 |
| Healthcare | €1,145.8 | €1,145.9 |
| Environment | €4,122,3 | €4,122,3 |
| Labour productivity | €53.0 | €323.2 |
| Labour participation | €356.3 | €2,100.7 |
| Policy revenue | €25,351.5 | €25,351.7 |
| Consumer surplus | €-26,540.7 | €-26,540.2 |
| VAT revenue | €-897.4 | €-897.5 |
| Policy implementation | €-20.0 | €-20.0 |
| **Total** | **€4,634.9** | **€8,484.3** |

**Table 7b.** Total welfare effect with discount rate of 1.5% – 30% meat tax. All values are in million 2018 euros.

| **Scenario 30% meat tax** | **Confidence interval low** | **Confidence interval high** |
| --- | --- | --- |
| Health - Low QALY | €2,037.0 | €5,534.2 |
| Healthcare | €2,189,9 | €2,189,7 |
| Environment | €7,705.4 | €7,705.4 |
| Labour productivity | €102.3 | €617.2 |
| Labour participation | €686.01 | €4,008.7 |
| Policy revenue | €46,570.4 | €46,570.4 |
| Consumer surplus | €-51,015.1 | €-51,015.1 |
| VAT revenue | €-1,677.6 | €-1,677.6 |
| Policy implementation | €-20.0 | €-20.0 |
| **Total** | **€6,578.2** | **€13,912.9** |

**Table 7c.** Total welfare effect with discount rate of 1.5% – 10% fruit and vegetable subsidy. All values are in million 2018 euros.

| **Scenario 10% subsidy** | **Confidence interval low** | **Confidence interval high** |
| --- | --- | --- |
| Health - Low QALY | €1,345.6 | €2,020.1 |
| Healthcare | €791.2 | €791.2 |
| Environment | €-137.7 | €-137.7 |
| Labour productivity | €101.4 | €205.4 |
| Labour participation | €520.3 | €1,119.0 |
| Policy revenue | €-15,429.4 | €-15,429.4 |
| Consumer surplus | €12,319.1 | €12,319.1 |
| VAT revenue | €277.0 | €277.0 |
| Policy implementation | €-20.0 | €-20.0 |
| **Total** | **€2,614** | **€4,001** |

**Discount rate – 4%**

**Table 8a.** Total welfare effect with discount rate of 4% – 15% meat tax. All values are in million 2018 euros.

| **Scenario 15% meat tax** | **Confidence interval low** | **Confidence interval high** |
| --- | --- | --- |
| Health - Low QALY | €716.0 | €1,911.6 |
| Healthcare | €739,1 | €739,1 |
| Environment | €3,005.5 | €3,005.5 |
| Labour productivity | €34.9 | €217.3 |
| Labour participation | €229.9 | €1,334.6 |
| Policy revenue | €17,998.4 | €17,998.4 |
| Consumer surplus | €-18,842.5 | €-18,842.4 |
| VAT revenue | €-637.0 | €-637.0 |
| Policy implementation | €-20.0 | €-20.0 |
| **Total** | **€3,224.3** | **€5,707.1** |

**Table 8b.** Total welfare effect with discount rate of 4% – 30% meat tax. All values are in million 2018 euros.

| **Scenario 30% meat tax** | **Confidence interval low** | **Confidence interval high** |
| --- | --- | --- |
| Health - Low QALY | €1,371.5 | €3,651.0 |
| Healthcare | €1,412,1 | €1,412,1 |
| Environment | €5,618.9 | €5,618.9 |
| Labour productivity | €67.3 | €414.9 |
| Labour participation | €442.8 | €2,547.3 |
| Policy revenue | €46,570.4 | €46,570.4 |
| Consumer surplus | €-51,015.1 | €-51,015.1 |
| VAT revenue | €-1,149.6 | €-1,149.6 |
| Policy implementation | €-20.0 | €-20.0 |
| **Total** | **€3,298.4** | **€8,030.0** |

**Table 8c.** Total welfare effect with discount rate of 4% – 10% fruit and vegetable subsidy. All values are in million 2018 euros.

| **Scenario 10% subsidy** | **Confidence interval low** | **Confidence interval high** |
| --- | --- | --- |
| Health - Low QALY | €887.9 | €1,330.0 |
| Healthcare | €505,7 | €505,7 |
| Environment | €-100.8 | €-100.8 |
| Labour productivity | €65.6 | €133.9 |
| Labour participation | €332.7 | €712.5 |
| Policy revenue | €-8,832.0 | €-8,832.0 |
| Consumer surplus | €8,641.1 | €8,641.1 |
| VAT revenue | €194.6 | €194.6 |
| Policy implementation | €-20.0 | €-20.0 |
| **Total** | **€1,674.7** | **€2,564.9** |

**Table 9.** Net societal welfare for all the sensitivity analyses compared to the reference no tax or subsidy scenario.

| **Total welfare** | **15% meat tax** | | **30% meat tax** | | **10% fruits and**  **vegetables subsidy** | |
| --- | --- | --- | --- | --- | --- | --- |
|  | **QALY €50,000** | **QALY €100,000** | **QALY €50,000** | **QALY €100,000** | **QALY €50,000** | **QALY €100,000** |
| *Total welfare main model* | *€3,069 - 7,386* | *€3,904 - 9,632* | *€4,050 - 12,276* | *€5,648 - 16,565* | *€1,800 - 3,289* | *€2,842 - 4,853* |
| Discount 1.5% | €4,635 - 8,484 | €5,699 - 11,383 | €6,578 - 13,913 | €8,615 - 19,447 | €2,606 - 3,983 | €3,952 - 6,003 |
| Discount 4% | €2,300 - 5,707 | €3,940 - 7,619 | €3,298 - 8,030 | €4,670 - 11,681 | €1,675 - 2,565 | €2,563 - 1,235 |
| Friction cost | €2,060 - 6,377 | €2,895 - 8,623 | €2,116 - 10,342 | €3,720 - 14,672 | €1,111 - 2,601 | €2,154 - 4,165 |
| HEC-LEG | €5,296 - 9,613 | €6,131 - 11,859 | €6,898 - 15,124 | €8,496 - 19,413 | €1,690 - 3,180 | €2,733 - 4,743 |
| LEC-HEG | €536 - 4,853 | €1,371 - 7,099 | €-141 - 8,085 | €1,457 - 12,374 | €1,887 - 3,377 | €2,930 - 4,941 |
| High price elasticity | €3,678 - 8,013 | €4,699 - 10,445 | €4,505 - 12,765 | €6,261 - 17,213 | €2,159 - 3,656 | €3,377 - 5,394 |
| Low price elasticity | €2,648 - 6,983 | €3,360 - 9,107 | €3,408 - 11,669 | €4,774 - 15,725 | €1,512 - 3,008 | €2,357 - 4,375 |
| Perfect Information | €2,235 - 5,140 | €2,235 - 5,140 | €2,452 - 7,988 | €2,452 - 7,988 | €757 - 1,726 | €757 - 1,726 |

Values are expressed in million 2018 euros.

HEC-LEG: High environmental costs at low efficiency gain in production over time.

LEC-HEG: Low environmental costs at high efficiency gain in production over time.

Price elasticity low: -0.54 for meat and -0.48 for fruit and vegetables.

Price elasticity high: -0.66 for meat and -0.59 for fruit and vegetables.

Perfect information: gained or lost health because of consumption already accounted for by consumers; QALYs not considered in total welfare.

**References**

1. Devisch I. Food taxes: a new holy grail? International journal of health policy and management. 2013;1(2):95.

2. Romijn G, Renes G. Algemene leidraad voor maatschappelijke kosten-batenanalyse2013.

3. Koopmans C, Heyma A, Hof B, Imandt M, Kok L, Pomp M. Werkwijzer voor kosten-batenanalyse in het sociale domein. Amsterdam: SEO Economisch Onderzoek; 2016. Report No.: SEO-rapport nr. 2016-11A Contract No.: ISBN 978-90-6733-805-9.

4. Bruyn Sd, Blom M, Schep E, Warringa G. CE_Delft_7A76_Werkwijzer_voor_MKBAs_DEF. 2017.

5. Cost of illness 2015 [Internet]. RIVM. 2018 [cited 23-08-2018]. Available from: <https://www.volksgezondheidenzorg.info/kosten-van-ziekten>.

6. Donker MDG. NIVEL Primary Care Database-Sentinel Practices. 2016.

7. Poortvliet M, Schrijvers C, Baan C. Diabetes in Nederland. Omvang, risicofactoren en gevolgen, nu en in de toekomst. 2007.

8. Hopman P, Gijsen B, Brink M, Rijken M. Zorg-en leefsituatie van mensen met kanker 2012. Deelrapportage I: Ervaringen met ziekenhuiszorg NIVEL. 2012.

9. Besseling J, de Vromme E, Hesselink JK, Sanders J. Arbeidsparticipatie van arbeidsgehandicapten: Den Haag: SCP; 2007.

10. Van Velzen J, Van Bennekom C, Edelaar M, Sluiter JK, Frings-Dresen M. How many people return to work after acquired brain injury?: a systematic review. Brain injury. 2009;23(6):473-88.

11. voor de Statistiek CB. Statline: Centraal Bureau voor de Statistiek; 1997.

12. Eurostat. Minimum wage statistics. 2017.

13. Loeppke R, Taitel M, Haufle V, Parry T, Kessler RC, Jinnett K. Health and productivity as a business strategy: a multiemployer study. Journal of Occupational and Environmental Medicine. 2009;51(4):411-28.

14. Balasooriya-Smeekens C, Bateman A, Mant J, De Simoni A. Barriers and facilitators to staying in work after stroke: insight from an online forum. BMJ Open. 2016;6(4):e009974.

15. Andersen G, Christensen D, Kirkevold M, Johnsen S. Post‐stroke fatigue and return to work: a 2‐year follow‐up. Acta Neurologica Scandinavica. 2012;125(4):248-53.

16. Terluin I, Verhoog A, Dagevos J, Van Horne P, R H. Vleesconsumptie per hoofd van de bevolking in Nederland, 2005-2016. Wageningen Economic Research; 2017.

17. Ma S, Kockelman KM, Fagnant DJ. Welvare analysis using logsum differences vs. rule of half: a series of cases studies 2. Transportation Research. 2015.

18. Wit GA, Gils PF, Over E, Suijkerbuijk A, Lokkerbol J, Smit H-FE, et al. Maatschappelijke kosten-baten analyse van beleidsmaatregelen om alcoholgebruik te verminderen2016.

19. Hakkaart-van Roijen L, Van der Linden N, Bouwmans C, Kanters T, Swan Tan S. Kostenhandleiding: methodologie van kostenonderzoek en referentieprijzen voor economische evaluaties in de gezondheidszorg. Institute for Medical Technology Assessment of Erasmus Universiteit Rotterdam; 2015.
