# Supplementary material for: A social cost-benefit analysis of meat taxation and a fruit and vegetables subsidy for a healthy and sustainable food consumption in the Netherlands
Source: BMC Public Health. 2020 May 11;20:643. doi: 10.1186/s12889-020-08590-z (PMC7212616; doi:10.1186/s12889-020-08590-z)
Supplement: Supplementary file 2 — Additional file 2. Health impact assessment. [file 12889_2020_8590_MOESM2_ESM.docx]

**Supplemental file 2. Health impact assessment.**

**DYNAMO-HIA model and data input**

The DYNAMO-HIA (Dynamic Modelling for Health Impact Analysis, https://www.dynamo-hia.eu/en) model was used to assess the health impact of the selected scenarios. DYNAMO-HIA is a Markov-type model that combines microsimulation of the risk factor exposure and macro simulation of the disease and survival. The DYNAMO-HIA model simulates individuals and their risk factor biographies of every year, given age- and sex-specific transition risks within that risk factor. The risk factor status determines the relative risk of a person to contract a disease or to die. Transition probabilities of each individual is updated in annual increments, **Figure 1** presents a stylized structure of the macro simulation as used in DYNAMO-HIA (1). Validation of the model was discussed by Lhachimi *et al. (2)*. A more detailed description of the model was published by Boshuizen *et al*. (1), version 2.0.8 was used in the present study.


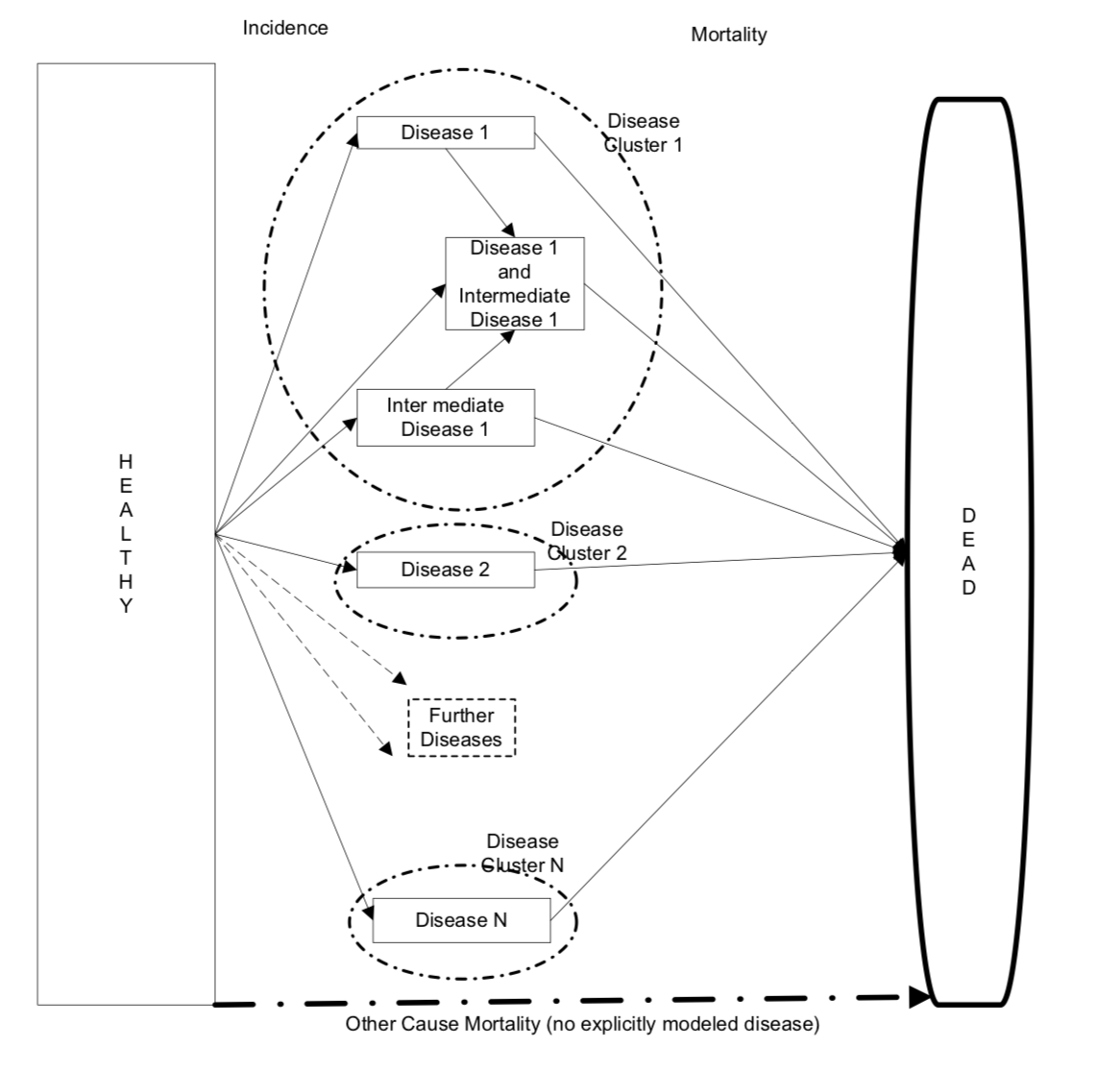


**Figure 1**. Stylized structure of disease life table used in DYNAMO-HIA (1).

**Population**

Dutch population data was obtained from the DYNAMO-HIA website and included data on newborns, size, overall Disability Adjusted Life Years (DALY) weights, overall disability, and overall mortality. Data collection was performed in 2011, and files were previously used in the DYNAMO-HIA modelling for the Public Health Foresight Study of the Netherlands in 2014 (VTV-2014). Within the simulations of this study, newborns were included in our simulation.

**Diseases**

A detailed description of data collection of the excess mortality and disability weights of the modelled diseases can be found on the DYNAMO-HIA website: https://www.dynamo-hia.eu; Documents and publications. Prevalence and incidence of the modelled diseases in the Netherlands, based on 2011 data, were used in this study and can also be found on the website.

**Risk factors**

Included risk factors were meat in one model, and fruit and vegetables (F&V) in another model. Disease relative risks associated with these food groups were derived from a 2015 systematic literature review of the Dutch Health Council. We used only the significant relative risks from the report of the Dutch Health Council (3, 4). Although total meat is taxed in our scenarios, only the consumption of red and processed meat in associated with increased risk of several diseases (e.g. stroke, diabetes, lung cancer, and colorectal cancer). Therefore, in the DYNAMO-HIA model only these meat types were considered. Relative risks of F&V were pooled to estimate health effects.

Consumption of red meat, processed meat, poultry, and F&V was based on data from the Dutch National Food Consumption Survey 2012-2014 and used as the baseline values in 2018. Food intake data was collected among a representative sample of the population living in the Netherlands on two non-consecutive days with 24hr dietary recalls conducted by dieticians (5). Individual data of red meat and processed meat were aggregated to calculate meat intake values (**Table 1**). For meat consumption (without poultry), categories were created per 50 grams. For fruit and vegetable consumption, categories were created per 150 grams. Additionally, in both consumption groups a 0 category was added. Net transition probabilities between consumption categories were used to estimate the effects of future consumption. Net-transition retain the age-specific risk-factor prevalence constant, effectively assuming that consumption patterns per age category will remain similar over time. This approach was chosen due to uncertainty of presence and direction of an autonomous trend in consumption in the Netherlands, as shown by the recent Dutch national food consumption survey and a yearly report on meat consumption in the Netherlands by Wageningen Economic Research (6-8). Price elasticity of demand estimates were obtained from a systematic literature review (**Table 1**, (9)).

**Table 1.** Included food groups and GloboDiet classifications.

| **Product** | **GloboDiet classification** | **Definition(3, 4)** | **Price elasticity (9)** |
| --- | --- | --- | --- |
| Meat | 0701  0702  0704  0705 | Red meat that is only cut or is prepared in the form of minced meat (beef, pig, sheep/lamb, horse and goat meat). Processed meat is meat that has been smoked or salted for preservation purposes or if preservatives have been added to it. Processed meat can be both white and red. White meat (poultry), includes chicken, turkey, and duck. | -0.60 (-0.66 to -0.54) |
| Fruit and  vegetables | 02  0401 | Green leafy vegetables, fruit-bearing vegetables (including peppers and tomatoes), root crops, cabbage crops, onion and garlic, stem crops and other vegetables. Fruit is defined as citrus fruit and non-citrus fruit. Fresh as well as dried and canned fruit are included. | -0.53 (-0.59 to -0.48) |

Consumption over time was modelled using DYNAMO-HIA. To also consider changes in the consumption of poultry, the ratio poultry to red & processed meat at baseline was used.

**Estimated prevented cases compared to reference scenario**

We performed probabilistic sensitivity analyses (PSA) on both the meat and fruit and vegetable models to estimate uncertainties of model estimates. We used the upper and lower estimates of the relative risks of consumption of the individual disease, derived from the systematic literature reviews and assumed the relative risks to be normally distributed. We applied 100 iterations of the model using Monte Carlo simulations. Only the upper and lower limit of prevented cases are used in the SCBA and presented in **Table 2, 3, and 4**. In **Table 5**, the gained Quality Adjusted Life Years (QALYs) of the scenarios are presented.

**Table 2.** Estimated difference in cases per year in a 15% meat tax scenario compared to the reference scenario based on the 95% Confidence Intervals range of the risk factors. Only the upper (UL) and lower limit (LL) of prevented cases are used in the SCBA and presented.

|  | **Estimated cases prevented compared to reference scenario** | | | | | | | | | |
| --- | --- | --- | --- | --- | --- | --- | --- | --- | --- | --- |
|  | **Colorectal cancer** | | **Diabetes** | | **CHD** | | **Lung cancer** | | **Stroke** | |
| **Year** | **LL** | **UL** | **LL** | **UL** | **LL** | **UL** | **LL** | **UL** | **LL** | **UL** |
| 2019 | 0 | -160 | -194 | -862 | 5 | 13 | -73 | -195 | -63 | -210 |
| 2020 | -2 | -308 | -378 | -1,703 | 13 | 32 | -127 | -340 | -122 | -412 |
| 2021 | -5 | -443 | -552 | -2,525 | 23 | 56 | -167 | -449 | -179 | -605 |
| 2022 | -8 | -569 | -720 | -3,331 | 32 | 84 | -198 | -534 | -233 | -790 |
| 2023 | -12 | -685 | -881 | -4,119 | 42 | 114 | -223 | -602 | -284 | -969 |
| 2024 | -17 | -794 | -1,035 | -4,887 | 52 | 146 | -243 | -657 | -333 | -1,141 |
| 2025 | -22 | -895 | -1,183 | -5,637 | 61 | 180 | -260 | -705 | -381 | -1,306 |
| 2026 | -28 | -989 | -1,324 | -6,364 | 69 | 214 | -275 | -745 | -426 | -1,464 |
| 2027 | -34 | -1,077 | -1,460 | -7,068 | 76 | 248 | -287 | -781 | -469 | -1,616 |
| 2028 | -40 | -1,158 | -1,589 | -7,750 | 82 | 282 | -299 | -814 | -510 | -1,761 |
| 2029 | -46 | -1,235 | -1,713 | -8,407 | 87 | 316 | -309 | -844 | -549 | -1,900 |
| 2030 | -52 | -1,307 | -1,830 | -9,041 | 92 | 350 | -319 | -871 | -586 | -2,033 |
| 2031 | -59 | -1,374 | -1,942 | -9,650 | 95 | 383 | -328 | -898 | -622 | -2,160 |
| 2032 | -66 | -1,438 | -2,047 | -10,232 | 99 | 416 | -336 | -923 | -656 | -2,281 |
| 2033 | -73 | -1,497 | -2,146 | -10,787 | 101 | 449 | -344 | -946 | -688 | -2,395 |
| 2034 | -80 | -1,551 | -2,238 | -11,312 | 103 | 480 | -351 | -968 | -718 | -2,503 |
| 2035 | -87 | -1,601 | -2,323 | -11,803 | 105 | 511 | -357 | -987 | -746 | -2,604 |
| 2036 | -94 | -1,647 | -2,401 | -12,262 | 107 | 541 | -363 | -1,003 | -771 | -2,697 |
| 2037 | -102 | -1,688 | -2,474 | -12,691 | 108 | 569 | -367 | -1,019 | -794 | -2,783 |
| 2038 | -109 | -1,726 | -2,539 | -13,088 | 110 | 597 | -372 | -1,033 | -815 | -2,861 |
| 2039 | -116 | -1,760 | -2,599 | -13,455 | 111 | 623 | -376 | -1,046 | -835 | -2,933 |
| 2040 | -123 | -1,791 | -2,653 | -13,791 | 112 | 648 | -379 | -1,058 | -852 | -2,998 |
| 2041 | -130 | -1,817 | -2,701 | -14,095 | 114 | 671 | -382 | -1,067 | -866 | -3,055 |
| 2042 | -137 | -1,839 | -2,743 | -14,368 | 115 | 693 | -384 | -1,075 | -879 | -3,105 |
| 2043 | -143 | -1,859 | -2,781 | -14,613 | 116 | 712 | -386 | -1,082 | -890 | -3,149 |
| 2044 | -150 | -1,874 | -2,813 | -14,829 | 117 | 730 | -387 | -1,088 | -899 | -3,186 |
| 2045 | -155 | -1,888 | -2,842 | -15,021 | 118 | 747 | -389 | -1,093 | -907 | -3,219 |
| 2046 | -161 | -1,898 | -2,866 | -15,187 | 120 | 762 | -389 | -1,096 | -913 | -3,245 |
| 2047 | -166 | -1,907 | -2,887 | -15,330 | 121 | 775 | -390 | -1,099 | -918 | -3,267 |
| 2048 | -170 | -1,912 | -2,903 | -15,449 | 123 | 787 | -390 | -1,100 | -921 | -3,283 |

**Table 3.** Estimated difference in cases per year in a 30% meat tax scenario compared to the reference scenario based on the 95% Confidence Intervals range of the risk factors. Only the upper (UL) and lower limit (LL) of prevented cases are used in the SCBA and presented.

|  | **Estimated cases prevented compared to reference scenario** | | | | | | | | | |
| --- | --- | --- | --- | --- | --- | --- | --- | --- | --- | --- |
|  | **Colorectal cancer** | | **Diabetes** | | **CHD** | | **Lung cancer** | | **Stroke** | |
| **Year** | **LL** | **UL** | **LL** | **UL** | **LL** | **UL** | **LL** | **UL** | **LL** | **UL** |
| 2019 | 0 | -306 | -372 | -1,643 | 9 | 23 | -139 | -370 | -120 | -398 |
| 2020 | -3 | -587 | -727 | -3,252 | 24 | 57 | -242 | -645 | -234 | -781 |
| 2021 | -7 | -848 | -1,065 | -4,826 | 40 | 102 | -320 | -854 | -342 | -1,150 |
| 2022 | -13 | -1,090 | -1,388 | -6,368 | 58 | 153 | -381 | -1,016 | -447 | -1,506 |
| 2023 | -20 | -1,314 | -1,698 | -7,874 | 75 | 210 | -428 | -1,145 | -546 | -1,849 |
| 2024 | -28 | -1,523 | -1,995 | -9,343 | 92 | 270 | -467 | -1,252 | -642 | -2,179 |
| 2025 | -37 | -1,718 | -2,280 | -10,776 | 108 | 333 | -500 | -1,342 | -733 | -2,497 |
| 2026 | -47 | -1,900 | -2,552 | -12,165 | 122 | 397 | -528 | -1,420 | -821 | -2,802 |
| 2027 | -58 | -2,069 | -2,812 | -13,511 | 135 | 463 | -552 | -1,488 | -904 | -3,094 |
| 2028 | -69 | -2,226 | -3,059 | -14,809 | 146 | 528 | -574 | -1,548 | -984 | -3,373 |
| 2029 | -81 | -2,373 | -3,294 | -16,060 | 156 | 594 | -593 | -1,603 | -1,059 | -3,639 |
| 2030 | -93 | -2,510 | -3,517 | -17,262 | 164 | 659 | -611 | -1,655 | -1,131 | -3,892 |
| 2031 | -105 | -2,639 | -3,728 | -18,417 | 171 | 724 | -627 | -1,704 | -1,200 | -4,134 |
| 2032 | -118 | -2,759 | -3,928 | -19,521 | 178 | 787 | -643 | -1,749 | -1,264 | -4,363 |
| 2033 | -132 | -2,871 | -4,115 | -20,568 | 183 | 850 | -657 | -1,791 | -1,324 | -4,579 |
| 2034 | -145 | -2,975 | -4,290 | -21,562 | 187 | 911 | -670 | -1,831 | -1,381 | -4,783 |
| 2035 | -159 | -3,071 | -4,453 | -22,498 | 191 | 971 | -682 | -1,868 | -1,433 | -4,973 |
| 2036 | -173 | -3,159 | -4,604 | -23,378 | 195 | 1,029 | -693 | -1,902 | -1,482 | -5,150 |
| 2037 | -187 | -3,240 | -4,743 | -24,200 | 199 | 1,084 | -703 | -1,933 | -1,526 | -5,313 |
| 2038 | -201 | -3,314 | -4,870 | -24,962 | 203 | 1,138 | -712 | -1,961 | -1,566 | -5,464 |
| 2039 | -214 | -3,380 | -4,985 | -25,664 | 207 | 1,189 | -720 | -1,986 | -1,602 | -5,600 |
| 2040 | -228 | -3,439 | -5,089 | -26,306 | 211 | 1,237 | -726 | -2,008 | -1,635 | -5,723 |
| 2041 | -242 | -3,491 | -5,181 | -26,887 | 215 | 1,282 | -732 | -2,026 | -1,663 | -5,832 |
| 2042 | -255 | -3,535 | -5,263 | -27,409 | 219 | 1,324 | -736 | -2,042 | -1,687 | -5,927 |
| 2043 | -267 | -3,572 | -5,334 | -27,873 | 222 | 1,363 | -739 | -2,054 | -1,707 | -6,010 |
| 2044 | -279 | -3,602 | -5,395 | -28,282 | 226 | 1,398 | -741 | -2,063 | -1,724 | -6,080 |
| 2045 | -290 | -3,625 | -5,447 | -28,635 | 229 | 1,430 | -742 | -2,069 | -1,737 | -6,138 |
| 2046 | -300 | -3,642 | -5,490 | -28,938 | 233 | 1,458 | -742 | -2,072 | -1,747 | -6,185 |
| 2047 | -310 | -3,655 | -5,525 | -29,192 | 236 | 1,484 | -741 | -2,074 | -1,755 | -6,222 |
| 2048 | -319 | -3,662 | -5,551 | -29,398 | 240 | 1,507 | -740 | -2,072 | -1,759 | -6,249 |

**Table 4.** Estimated difference in cases per year in a 10% fruit and vegetables subsidy scenario compared to the reference scenario based on the 95% Confidence Intervals range of the risk factors. Only the upper (UL) and lower limit (LL) of prevented cases are used in the SCBA and presented.

|  | **Estimated cases prevented compared to reference scenario** | | | | | | | | | |
| --- | --- | --- | --- | --- | --- | --- | --- | --- | --- | --- |
|  | **Colorectal cancer** | | **Diabetes** | | **CHD** | | **Lung cancer** | | **Stroke** | |
| **Year** | **LL** | **UL** | **LL** | **UL** | **LL** | **UL** | **LL** | **UL** | **LL** | **UL** |
| 2019 | -30 | -63 | -37 | -126 | -132 | -224 | -18 | -34 | -120 | -228 |
| 2020 | -57 | -121 | -70 | -248 | -260 | -443 | -31 | -60 | -234 | -447 |
| 2021 | -82 | -175 | -100 | -364 | -384 | -655 | -41 | -78 | -344 | -657 |
| 2022 | -104 | -224 | -127 | -476 | -504 | -862 | -48 | -93 | -448 | -857 |
| 2023 | -125 | -269 | -151 | -584 | -621 | -1,063 | -54 | -104 | -548 | -1,049 |
| 2024 | -143 | -311 | -173 | -688 | -733 | -1,258 | -58 | -113 | -643 | -1,233 |
| 2025 | -160 | -350 | -193 | -788 | -843 | -1,448 | -62 | -121 | -735 | -1,409 |
| 2026 | -176 | -386 | -211 | -885 | -948 | -1,632 | -65 | -128 | -822 | -1,578 |
| 2027 | -190 | -420 | -227 | -978 | -1,051 | -1,811 | -67 | -133 | -906 | -1,741 |
| 2028 | -204 | -452 | -241 | -1,066 | -1,149 | -1,983 | -69 | -139 | -986 | -1,896 |
| 2029 | -216 | -482 | -253 | -1,151 | -1,244 | -2,149 | -71 | -144 | -1,062 | -2,045 |
| 2030 | -227 | -510 | -264 | -1,232 | -1,335 | -2,309 | -73 | -148 | -1,136 | -2,188 |
| 2031 | -238 | -536 | -273 | -1,308 | -1,421 | -2,461 | -75 | -152 | -1,205 | -2,323 |
| 2032 | -247 | -560 | -280 | -1,380 | -1,502 | -2,605 | -76 | -156 | -1,271 | -2,452 |
| 2033 | -256 | -583 | -286 | -1,449 | -1,580 | -2,743 | -77 | -159 | -1,334 | -2,576 |
| 2034 | -264 | -604 | -290 | -1,513 | -1,653 | -2,872 | -78 | -163 | -1,393 | -2,692 |
| 2035 | -271 | -623 | -293 | -1,572 | -1,720 | -2,993 | -79 | -165 | -1,447 | -2,800 |
| 2036 | -277 | -641 | -294 | -1,627 | -1,782 | -3,105 | -80 | -168 | -1,498 | -2,900 |
| 2037 | -282 | -656 | -295 | -1,677 | -1,840 | -3,208 | -80 | -170 | -1,545 | -2,994 |
| 2038 | -286 | -670 | -294 | -1,723 | -1,892 | -3,302 | -80 | -171 | -1,588 | -3,080 |
| 2039 | -290 | -683 | -292 | -1,765 | -1,939 | -3,389 | -81 | -173 | -1,628 | -3,159 |
| 2040 | -293 | -695 | -290 | -1,805 | -1,983 | -3,469 | -81 | -175 | -1,664 | -3,233 |
| 2041 | -295 | -705 | -287 | -1,840 | -2,022 | -3,541 | -81 | -176 | -1,697 | -3,300 |
| 2042 | -297 | -714 | -284 | -1,871 | -2,056 | -3,605 | -81 | -177 | -1,726 | -3,359 |
| 2043 | -298 | -721 | -280 | -1,899 | -2,085 | -3,660 | -81 | -178 | -1,751 | -3,411 |
| 2044 | -299 | -726 | -276 | -1,923 | -2,110 | -3,707 | -81 | -178 | -1,772 | -3,455 |
| 2045 | -299 | -731 | -272 | -1,945 | -2,132 | -3,748 | -81 | -178 | -1,791 | -3,494 |
| 2046 | -299 | -735 | -269 | -1,965 | -2,151 | -3,785 | -81 | -179 | -1,807 | -3,529 |
| 2047 | -299 | -739 | -267 | -1,984 | -2,167 | -3,817 | -81 | -179 | -1,822 | -3,560 |
| 2048 | -299 | -741 | -264 | -2,000 | -2,181 | -3,844 | -80 | -180 | -1,834 | -3,586 |

**Table 5.** Change in Quality Adjusted Life Years of the three scenarios compared to the reference scenario. The lower limit (LL) and upper limit (UL) of the QALYs are presented.

| **QALYs gained** | **15% meat tax** | |  | **30% meat tax** | |  | **10% fruit and vegetable subsidy** | |
| --- | --- | --- | --- | --- | --- | --- | --- | --- |
| *Year* | **LL** | **UL** |  | **LL** | **UL** |  | **LL** | **UL** |
| 2019 | 145 | 296 |  | 276 | 564 |  | 135 | 195 |
| 2020 | 269 | 564 |  | 515 | 1,078 |  | 260 | 378 |
| 2021 | 377 | 811 |  | 723 | 1,552 |  | 376 | 549 |
| 2022 | 472 | 1,041 |  | 908 | 1,994 |  | 484 | 709 |
| 2023 | 557 | 1,256 |  | 1,072 | 2,407 |  | 585 | 860 |
| 2024 | 634 | 1,459 |  | 1,220 | 2,797 |  | 681 | 1,003 |
| 2025 | 703 | 1,650 |  | 1,353 | 3,165 |  | 771 | 1,138 |
| 2026 | 764 | 1,831 |  | 1,473 | 3,511 |  | 856 | 1,265 |
| 2027 | 821 | 2,001 |  | 1,581 | 3,838 |  | 936 | 1,387 |
| 2028 | 872 | 2,162 |  | 1,678 | 4,144 |  | 1,011 | 1,501 |
| 2029 | 918 | 2,313 |  | 1,765 | 4,432 |  | 1,081 | 1,608 |
| 2030 | 959 | 2,456 |  | 1,843 | 4,701 |  | 1,148 | 1,710 |
| 2031 | 997 | 2,590 |  | 1,913 | 4,954 |  | 1,209 | 1,804 |
| 2032 | 1,030 | 2,715 |  | 1,974 | 5,189 |  | 1,265 | 1,891 |
| 2033 | 1,060 | 2,831 |  | 2,028 | 5,405 |  | 1,318 | 1,973 |
| 2034 | 1,085 | 2,937 |  | 2,074 | 5,604 |  | 1,366 | 2,048 |
| 2035 | 1,105 | 3,033 |  | 2,113 | 5,784 |  | 1,409 | 2,116 |
| 2036 | 1,122 | 3,118 |  | 2,144 | 5,947 |  | 1,447 | 2,176 |
| 2037 | 1,135 | 3,194 |  | 2,169 | 6,093 |  | 1,481 | 2,230 |
| 2038 | 1,144 | 3,261 |  | 2,188 | 6,221 |  | 1,511 | 2,278 |
| 2039 | 1,151 | 3,320 |  | 2,201 | 6,331 |  | 1,536 | 2,320 |
| 2040 | 1,155 | 3,370 |  | 2,208 | 6,426 |  | 1,559 | 2,357 |
| 2041 | 1,156 | 3,411 |  | 2,209 | 6,503 |  | 1,578 | 2,388 |
| 2042 | 1,155 | 3,444 |  | 2,206 | 6,566 |  | 1,593 | 2,414 |
| 2043 | 1,152 | 3,471 |  | 2,199 | 6,615 |  | 1,604 | 2,434 |
| 2044 | 1,147 | 3,491 |  | 2,188 | 6,651 |  | 1,612 | 2,448 |
| 2045 | 1,141 | 3,507 |  | 2,175 | 6,676 |  | 1,618 | 2,460 |
| 2046 | 1,134 | 3,518 |  | 2,159 | 6,690 |  | 1,623 | 2,471 |
| 2047 | 1,127 | 3,524 |  | 2,142 | 6,695 |  | 1,627 | 2,478 |
| 2048 | 1,118 | 3,525 |  | 2,122 | 6,691 |  | 1,629 | 2,483 |

**Sensitivity analysis**

We varied the price elasticity (PE) using the upper and lower estimate of the 95% confidence interval. Meat: -0.60 (95% CI: -0.66; -0.54) and F&V: -0.53 (95% CI: -0.59; -0.48). This changed price elasticity will affected consumption changes and therefore related health outcomes. **Tables 6, 7, and 8** present the estimated mean cases in the different PE scenarios as compared to the main analysis.

**Table 6**. One-way sensitivity analysis of price elasticity for the 15% meat tax scenario.

|  | **Sum estimated cases compared to reference scenario** | | |
| --- | --- | --- | --- |
| **Disease** | **Lower bound PE** | **Main analysis** | **Upper bound PE** |
| Colorectal cancer | -16,950 | -18,954 | -20,252 |
| Diabetes type 2 | -168,645 | -182,840 | -205,197 |
| CHD | 6,938 | 7,647 | 7,924 |
| Lung cancer | -16,009 | -17,392 | -19,509 |
| Stroke | -39,250 | -42,857 | -47,341 |
| QALYs | 48,548 | 52,718 | 58,998 |

**Table 7.** One-way sensitivity analysis of price elasticity for the 30% meat tax scenario.

|  | **Sum estimated cases compared to reference scenario** | | |
| --- | --- | --- | --- |
| **Disease** | **Lower bound PE** | **Main Analysis** | **Upper bound PE** |
| Colorectal cancer | -33,516 | -36,461 | -38,493 |
| Diabetes type 2 | -322,730 | -349,136 | -368,089 |
| CHD | 12,642 | 14,411 | 15,273 |
| Lung cancer | -30,589 | -33,075 | -35,351 |
| Stroke | -75,371 | -81,808 | -86,028 |
| QALYs | 93,587 | 101,465 | 106,721 |

**Table 8.** One-way sensitivity analysis of price elasticity for the 10% fruits and vegetables subsidy.

|  | **Sum estimated cases compared to reference scenario** | | |
| --- | --- | --- | --- |
| **Disease** | **Lower bound PE** | **Reference** | **Upper bound PE** |
| Colorectal cancer | -10,735 | -11,511 | -13,357 |
| Diabetes type 2 | -20,860 | -23,718 | -25,092 |
| CHD | -55,068 | -59,842 | -67,972 |
| Lung cancer | -2,965 | -3,176 | -3,668 |
| Stroke | -50,057 | -54,079 | -61,893 |
| QALYs | 37,698 | 44,375 | 50,282 |

**References**

Uncategorized References

1. Boshuizen HC, Lhachimi SK, van Baal PH, Hoogenveen RT, Smit HA, Mackenbach JP, et al. The DYNAMO-HIA model: an efficient implementation of a risk factor/chronic disease Markov model for use in Health Impact Assessment (HIA). Demography. 2012;49(4):1259-83.

2. Lhachimi SK, Nusselder WJ, Smit HA, van Baal P, Baili P, Bennett K, et al. DYNAMO-HIA–A Dynamic Modeling Tool for Generic Health Impact Assessments. PLOS ONE. 2012;7(5):e33317.

3. Gezondheidsraad. Vlees - Achtergronddocument bij Richtlijnen goede voeding 2015. Den Haag: Gezondheidsraad. 2015;publicatienr. A15/27.

4. Gezondheidsraad. Groente en fruit - Achtergronddocument bij Richtlijnen goede voeding 2015. Den Haag: Gezondheidsraad. 2015(A15/12).

5. Van Rossum C, Buurma-Rethans E, Vennemann F, Beukers M, Brants HA, De Boer E, et al. The diet of the Dutch: Results of the first two years of the Dutch National Food Consumption Survey 2012-2016. 2016.

6. Van de Kassteele J, Hoogenveen R, Engelfriet P, Van Baal P, Boshuizen H. Estimating net transition probabilities from cross‐sectional data with application to risk factors in chronic disease modeling. Statistics in medicine. 2012;31(6):533-43.

7. Van Rossum CTM, Buurma-Rethans EJM, Vennemann FBC, Beukers M, Brants HAM, Boer EJd, et al. The diet of the Dutch; Results of the first 2 year of the Dutch National Food Consumption Survey 2012-2014. Bilthoven: Rijksinstituut voor Volksgezondheid en Milieu (RIVM); 2016. Report No.: 2016-0082.

8. Terluin I, Verhoog A, Dagevos J, Van Horne P, R H. Vleesconsumptie per hoofd van de bevolking in Nederland, 2005-2016. Wageningen Economic Research; 2017.

9. Green R, Cornelsen L, Dangour AD, Turner R, Shankar B, Mazzocchi M, et al. The effect of rising food prices on food consumption: systematic review with meta-regression. Bmj. 2013;346:f3703.
