# Supplementary material for: A social cost-benefit analysis of meat taxation and a fruit and vegetables subsidy for a healthy and sustainable food consumption in the Netherlands
Source: BMC Public Health. 2020 May 11;20:643. doi: 10.1186/s12889-020-08590-z (PMC7212616; doi:10.1186/s12889-020-08590-z)
Supplement: Supplementary file 3 — Additional file 3. Estimating environmental impact of dietary changes. [file 12889_2020_8590_MOESM3_ESM.docx]

**Supplemental file 3. Estimating environmental impact of dietary changes.**

**Life Cycle Analyses**

The environmental impact of the Dutch food consumption was estimated with Life Cycle Analysis (LCA), a methodological tool to assess the environmental load throughout the life cycle of a product. The system boundaries of LCA were from cradle till plate, indicating all phases from primary production, processing, primary packaging, distribution, supermarket and transport through all phases (except from supermarket to consumer), storage, preparing, cooking and incineration of waste products. Life Cycle Inventories (LCI) data representative for Dutch situations were previously provided by Blonk Consultants in 2015-2018 and were saved in SimaPro. LCIs are all the in and out flows of a product system, including raw resources or materials, energy by type, water, and emissions to air, water and land. The LCI data was used by the RIVM to calculate the total environmental impact of the food consumption using the ReCiPe 2016 model [1]. The result is the average environmental impact per kg prepared food at the plate. The database included environmental impact data on the indicators land use (LU), greenhouse gas (GHG) emissions, acidification, and eutrophication of salt- and fresh water.

**Extrapolations**

Primary LCA data was available for 225 foods and drinks. Foods that were not covered were extrapolated as a proxy to primary LCA data. Extrapolations were done by an expert judgment of a panel of RIVM scientists including a cross check of each other’s extrapolations. The extrapolations were based on ingredient composition, similarities in production system, and similarities in type of food and variety. Conversion factors were applied to calculate the impact of a processed item (shrinking, losses, drying etc.). For example, a factor for grapes was used to calculate the impact of dried raisins based on the water content of both. Standardized recipes from the Dutch Food Composition Table (2016) were used to calculate the environmental load of composite dishes. The RIVM panel constructed other recipes based on literature, ingredients list and expert knowledge when no standardized recipes were available.

**Environmental impact of consumption**

Data of the Dutch National Food Consumption Survey (DNFCS, [2]) 2012-2014 was used to estimate daily consumptions of meat products, as well as fruit and vegetables. Both the DNFCS as well as the environmental impact data were available at food product level. The mean environmental impact for all five environmental impact indicators (for example, mean CO2-eq per consumed kg meat) in the DNFCS was calculated (**Table 1**).

Total estimated annual consumption of meat (white, red, and processed), and fruit and vegetables (F&V) for the Dutch population for the years 2018 until 2048 was derived from the Dynamo-HIA model (Supplemental file 1, [3]). The difference in annual total consumption was calculated comparing the scenarios (tax or subsidy) to a reference scenario. These estimated annual differences in consumption were then multiplied by the mean environmental impact (land use, greenhouse gas emissions, acidification, and eutrophication of salt- and fresh water).

**Table 1.** Mean environmental impact of a kilogram meat and fruit and vegetables.

| **Category** | **Unit** | **Meat** | **Fruit and vegetables** |
| --- | --- | --- | --- |
| Climate change | kg CO2-eq | 22.88 | 1.29 |
| Acidification | kg SO2-eq | 0.29 | 0.0036 |
| Eutrophication, fresh water | kg P-eq | 0.0047 | 0.0001 |
| Eutrophication, salt-water | kg N | 0.0493 | 0.0009 |
| Land use | m2a | 15.90 | 0.15 |

**Production efficiency gains**

It is expected that the environmental impact of food production per kg product will become lower (more efficient) over time. It was therefore important to take account of these expected production efficiency gains over time in the analysis. For the Netherlands, it was estimated that the agriculture and fishery sectors have reduced their standardized GHG emission by 20% in 16 years (1.25% per year, [4]), from 2000 to 2016. Therefore, in the main analysis we extrapolated this annual decrease up until 2048.

**Cost of environmental impact**

The environmental impact costs are described in a report of CE Delft [5]. The mean costs per enviromental indicator used in the Societal Cost Benefit Analysis (SCBA) are presented in **Table 2**. Cost levels in the SCBA are discounted by the recommended annual 3% to present all costs in 2018 Euro’s.

**Table 2.** Environmental impact costs according to CE Delft.

|  |  | **Environmental impact (external costs)** | | |
| --- | --- | --- | --- | --- |
| **Category** | **Unit** | **Low** | **Mean** | **High** |
| Climate change | €/kg CO2-eq | €0,014 | €0,057 | €0,057 |
| Acidification | €/kg SO2-eq | €1,19 | €5,4 | €10,7 |
| Eutrophication, fresh water | €/kg P-eq | €0,41 | €1,9 | €3,71 |
| Eutrophication, salt-water | €/kg N | €3,11 | €3,11 | €3,11 |
| Land use | €/m2a | €0,00647 | €0,0261 | €0,0507 |

**Costs of consumption**

In the main analysis of the SCBA the mean estimated annual efficiency gain in production over time of 1.25% per year was used to calculate the environmental impact of the changes in meat and F&V consumption. These estimated annual differences in consumption were then multiplied by the mean environmental impact (land use, greenhouse gas emissions, acidification, and eutrophication of salt- and fresh water).

**Sensitivity analyses**

In the sensitivity analyses, a high cost (Table 2) of the environmental indicators and a low efficiency gain in production (-0.75% per year) (HEC-LEG) was calculated to represent the upper bound of expected environmental costs/benefits (**Table 3**). In addition, an analysis with low environmental costs (Table 2) and high efficiency gains (-1.75% per year) (LEC-HEG) was performed which represents the lower bound of expected enviromental costs/benefits.

**Table 3**. Discounted differences in costs of all environmental impacts in the fruit and vegetables subsidy scenario and the meat taxation scenarios compared to a reference scenario^ab^.

| **Costs** | **Meat tax (15%)** | | | **Meat tax (30%)** | | | **Fruit and vegetables subsidy** | | |
| --- | --- | --- | --- | --- | --- | --- | --- | --- | --- |
| **Year** | **LEC – HEG** | **Main** | **HEC-LEG** | **LEC – HEG** | **Main** | **HEC-LEG** | **LEC – HEG** | **Main** | **HEC-LEG** |
| 2019 | -49.8 | -185.9 | -289.9 | -125.2 | -348.7 | -477.5 | 1.54 | 6.43 | 8.29 |
| 2020 | -47.9 | -179.4 | -281.3 | -120.1 | -336.0 | -462.31 | 1.47 | 6.17 | 8.22 |
| 2021 | -45.8 | -172.4 | -271.7 | -114.9 | -323.3 | -446.9 | 1.41 | 5.93 | 8.17 |
| 2022 | -43.9 | -166.3 | -263.34 | -110.2 | -311.6 | -432.8 | 1.34 | 5.70 | 8.11 |
| 2023 | -42.1 | -160.4 | -255.2 | -105.8 | -300.6 | -419.4 | 1.28 | 5.46 | 8.03 |
| 2024 | -40.4 | -154.6 | -247.4 | -101.1 | -288.8 | -404.98 | 1.23 | 5.26 | 7.99 |
| 2025 | -38.7 | -148.7 | -239.1 | -96.7 | -277.7 | -391.2 | 1.17 | 5.04 | 7.92 |
| 2026 | -37.0 | -142.8 | -230.7 | -92.5 | -266.9 | -377.8 | 1.12 | 4.84 | 7.85 |
| 2027 | -35.3 | -137.2 | -222.9 | -88.5 | -256.6 | -364.9 | 1.07 | 4.64 | 7.78 |
| 2028 | -33.8 | -132.1 | -215.5 | -84.5 | -246.4 | -352.0 | 1.02 | 4.44 | 7.68 |
| 2029 | -32.4 | -127.0 | -208.3 | -80.8 | -236.6 | -339.7 | 0.97 | 4.25 | 7.60 |
| 2030 | -31.0 | -122.4 | -201.8 | -77.4 | -227.9 | -328.8 | 0.92 | 4.07 | 7.53 |
| 2031 | -29.7 | -117.6 | -194.9 | -73.9 | -218.8 | -317.2 | 0.88 | 3.90 | 7.45 |
| 2032 | -28.3 | -112.8 | -187.9 | -70.7 | -210.4 | -306.4 | 0.84 | 3.73 | 7.36 |
| 2033 | -27.1 | -108.7 | -181.9 | -67.6 | -202.0 | -295.6 | 0.80 | 3.58 | 7.31 |
| 2034 | -25.9 | -104.2 | -175.3 | -64.56 | -194.0 | -285.3 | 0.76 | 3.43 | 7.24 |
| 2035 | -24.7 | -99.7 | -168.6 | -61.7 | -186.2 | -275.1 | 0.73 | 3.28 | 7.15 |
| 2036 | -23.5 | -95.5 | -162.2 | -58.8 | -178.4 | -264.8 | 0.69 | 3.13 | 7.06 |
| 2037 | -22.3 | -91.0 | -155.5 | -56.0 | -170.8 | -254.8 | 0.66 | 3.00 | 6.99 |
| 2038 | -21.1 | -86.7 | -148.8 | -53.3 | -163.5 | -245.0 | 0.63 | 2.87 | 6.89 |
| 2039 | -20.2 | -83.3 | -143.7 | -50.8 | -156.6 | -235.9 | 0.59 | 2.74 | 6.81 |
| 2040 | -19.2 | -79.6 | -138.0 | -48.4 | -150.0 | -227.0 | 0.57 | 2.63 | 6.74 |
| 2041 | -18.4 | -76.5 | -133.4 | -46.1 | -143.5 | -218.2 | 0.54 | 2.51 | 6.65 |
| 2042 | -17.5 | -73.3 | -128.4 | -43.8 | -137.2 | -209.6 | 0.51 | 2.39 | 6.57 |
| 2043 | -16.6 | -70.1 | -123.4 | -41.7 | -131.2 | -201.4 | 0.49 | 2.28 | 6.46 |
| 2044 | -15.8 | -66.9 | -118.5 | -39.7 | -125.4 | -193.3 | 0.46 | 2.18 | 6.38 |
| 2045 | -15.1 | -64.2 | -114.1 | -37.7 | -119.9 | -185.8 | 0.44 | 2.09 | 6.32 |
| 2046 | -14.4 | -61.5 | -109.9 | -35.8 | -114.3 | -178.0 | 0.42 | 2.00 | 6.26 |
| 2047 | -13.7 | -58.9 | -105.7 | -34.1 | -109.3 | -171.0 | 0.40 | 1.92 | 6.20 |
| 2048 | -13.0 | -56.3 | -101.6 | -32.4 | -104.4 | -164.1 | 0.38 | 1.83 | 6.13 |
| 2019 | -12.4 | -53.8 | -97.6 | -30.8 | -99.8 | -157.7 | 0.36 | 1.75 | 6.03 |
| **Total costs^c^** | **-857** | **-3,3890** | **-5,617** | **-2,146** | **-6,337** | **-9,184** | **25.7** | **113.5** | **223.2** |

a: discount rate 3%

b: cost levels of million 2018 Euro’s.

c: negative costs are benefits for society, positive costs are costs for society.

LEC-HEG: Low environmental impact indicators costs; high efficiency gains.

Main analysis: Medium environmental impact indicators costs; medium efficiency gains.

HEC-LEG: High environmental impact indicators costs; low efficiency gains.

**References**

1. Huijbregts, M., et al., *ReCiPe 2016: A harmonized life cycle impact assessment method at midpoint and endpoint level Report I: Characterization*. 2016, RIVM: Bilthoven.

2. Van Rossum, C.T.M., et al., *The diet of the Dutch; Results of the first 2 year of the Dutch National Food Consumption Survey 2012-2014.* 2016, Rijksinstituut voor Volksgezondheid en Milieu (RIVM): Bilthoven.

3. Boshuizen, H.C., et al., *The DYNAMO-HIA model: an efficient implementation of a risk factor/chronic disease Markov model for use in Health Impact Assessment (HIA).* Demography, 2012. **49**(4): p. 1259-1283.

4. Compendium voor de Leefomgeving. *Broeikasgas en CO2-intensiteit bedrijven, 1995-2016 (in Dutch)*. 2018 [cited 2018 13-11-2018]; Available from: <https://www.clo.nl/indicatoren/nl0542-broeikasgasintensiteit-bedrijven>.

5. De Bruyn, S., et al., *Environmental Prices Handbook 2017 - Methods and numbers for valuation of environmental impacts*. 2018, CE Delft: Delft.
